# A genome variation map provides insights into the genetics of walnut adaptation and agronomic traits

**DOI:** 10.1186/s13059-021-02517-6

**Published:** 2021-10-27

**Authors:** Feiyang Ji, Qingguo Ma, Wenting Zhang, Jie Liu, Yu Feng, Peng Zhao, Xiaobo Song, Jiaxin Chen, Junpei Zhang, Xin Wei, Ye Zhou, Yingying Chang, Pu Zhang, Xuehui Huang, Jie Qiu, Dong Pei

**Affiliations:** 1 State Key Laboratory of Tree Genetics and Breeding, Key Laboratory of Tree Breeding and Cultivation of the State Forestry and Grassland Administration, Research Institute of Forestry, Chinese Academy of Forestry, Beijing, 100091 China; 2 Shanghai Key Laboratory of Plant Molecular Sciences, College of Life Sciences, Shanghai Normal University, Shanghai, 200234 China; 3 Systematic & Evolutionary Botany and Biodiversity group, MOE Laboratory of Biosystem Homeostasis and Protection, College of Life Sciences, Zhejiang University, Hangzhou, 310058 Zhejiang China; 4 Key Laboratory of Resource Biology and Biotechnology in Western China, Ministry of Education, College of Life Sciences, Northwest University, Xi’an, 710069 Shaanxi China; 5 Engineering Laboratory of Biotechnology for Green Medicinal Plant of Henan Province, Engineering Technology Research Center of Nursing and Utilization of Genuine Chinese Crude Drugs of Colleges and Universities in Henan Province, College of Life Sciences, Henan Normal University, Xinxiang, 453007 Henan China

**Keywords:** Walnut, *Juglans regia*, Improvement, Adaptation, GWAS

## Abstract

**Background:**

Common walnut (*Juglans regia* L.) is one of the top four most consumed nuts in the world due to its health benefits and pleasant taste. Despite its economic importance, the evolutionary history and genetic control of its adaptation and agronomic traits remain largely unexplored.

**Results:**

We report a comprehensive walnut genomic variation map based on whole-genome resequencing of 815 walnut accessions. Evolutionary analyses suggest that Chinese *J. regia* diverged from *J. sigillata* with extensive hybridizations after the split of the two species. In contrast to annual crops, the genetic diversity and heterozygous deleterious mutations of Chinese common walnut trees have continued to increase during the improvement process. Selective sweep analyses identify 902 genes uniquely selected in the improved common walnut compared to its progenitor population. Five major-effect loci are identified to be involved in walnut adaptations to temperature, precipitation, and altitude. Genome-wide association studies reveal 27 genomic loci responsible for 18 important agronomic traits, among which *JrFAD2* and *JrANR* are the potentially major-effect causative genes controlling linoleic acid content and color of the endopleura of the nut, respectively.

**Conclusions:**

The largest genomic resource for walnuts to date has been generated and explored in this study, unveiling their evolutionary history and cracking the genetic code for agronomic traits and environmental adaptation of this economically crucial crop tree.

**Supplementary Information:**

The online version contains supplementary material available at 10.1186/s13059-021-02517-6.

## Background

The *Juglans* genus contains ~ 21 diploid species. Among them, common walnut (*Juglans regia* L.) is one of the top four most consumed nuts in the world. Rich in unsaturated fatty acids, proteins, minerals, and vitamins, the common walnut offers many benefits to human health. It is widely grown commercially in diverse temperate regions of Asia, Europe, North and South America, South Africa, Australia, and New Zealand. China is the world’s leading producer followed by the USA, Iran, and Turkey [1]. As the sister taxon of *J. regia*, *J. sigillata* harbors some wild nut characteristics, which may represent a primitive form, and is mainly distributed in southwest China [2]. A recent phylogenomic analysis revealed that *J. regia* and *J. sigillata* arose from an ancient hybridization that occurred between American black walnuts (*Rhysocaryon*) and Asian butternuts (*Cardiocaryon*) after a climate-driven range expansion in Eurasia during the Pliocene [3]. According to evidence from ^14^C-dated leaf fossils and carbonized nuts, *J. regia* has existed in China for more than 7000 years [4]. China is now regarded as one of the major centers of walnut genetic diversity, which form a rich germplasm source for walnut breeding [5]. However, knowledge of the population structure, genetic diversity, and the evolutionary history of the Chinese walnut remains limited [6].

The release of the first reference genome of *J. regia* by de novo assembly from short reads opened the genomic era for studies on walnut biology [7]. The quality of the *J. regia* reference genome has been improving, assisted by up-to-date long-read and Hi-C sequencing technologies [8, 9], which has laid a foundation for exploring the genetic mechanisms underlying desired agricultural traits of walnut, such as increased yield, larger nut size, ease of cracking, and light kernel color. In addition, for walnut and other economically important tree plants, the ability to adapt to specific climatic conditions as well as to climate change is also an important breeding priority. During recent years, researchers have been developing genomic tools for walnut breeding. For example, based on the resequencing data of 27 founders of the Walnut Improvement Program (WIP) of University of California, Davis, the *J. regia* 700 K SNP array was developed, which has greatly facilitated research and development in terms of walnut genetics and breeding [10]. Using this walnut panel, researchers undertook genome-wide association studies (GWAS) to map genomic loci associated with fruit phenotypes and traits related to phenology [11–13]. Some associated SNPs based on these findings were further applied to develop breeding markers for the selection of individuals with desirable traits [12]. However, due to limited variations within the 27 WIP walnut founders, many loci with minor allele frequency would inevitably be missed. In addition, while the associated loci could be identified based on an SNP array, it is often difficult to determine causative genes and variations responsible for the traits due to technical limits in the SNP array.

Population genomic analyses of seed-propagated annual crops have greatly advanced our understanding of plant domestication and evolution [14]. However, there is only limited knowledge about how genomes and genes change during the cultivation of long-lived perennial crop trees grown for their fruit [15–17]. In this study, we sequenced the genomes of a wide collection of walnut trees, including 815 *J. sigillata* and *J. regia* accessions from geographically diverse regions in China, as well as samples from Iran and Pakistan. Population structure, genetic diversity, and genetic load for Chinese walnut populations were investigated. Candidate genomic loci or genes involved in the improvement of Chinese walnuts have been identified based on genomic selective sweep analyses. In addition, potentially causative genes responsible for agronomic traits and their adaptations to multiple environmental factors were identified based on GWAS and genetic-environment association (GEA) analyses.

## Results

### Genome-wide variations and population structure of walnut population

We collected 815 *J. regia* and *J. sigillata* samples from China, Iran, and Pakistan. The Chinese accessions were sampled from 19 provinces covering most areas where common walnut is currently cultivated (Additional file 1: Table S1). We resequenced 8.4 Tb data for 815 walnut samples, with an average sequencing depth of ~ 19.0× for each accession. Reads were mapped to the *J. regia* reference genome [9], and the mapping rate was around 89.2% per sample (Additional file 1: Table S1). Through variant detection and filtering, 16.78 million SNPs were detected in the walnut genome. For each megabase window across the genome, the average number of SNPs is ~ 30.6 k for the 815 walnut individuals collected in this study (Additional file 2: Fig. S1). We performed Sanger sequencing to validate genotypes of 12–19 accessions for 74 SNP sites within 6 genomic loci (1232 SNP genotypes in total) and found the overall accuracy of the genotyping rate for the SNPs is 99.0% (Additional file 3: Table S2).

**Fig. 1:**
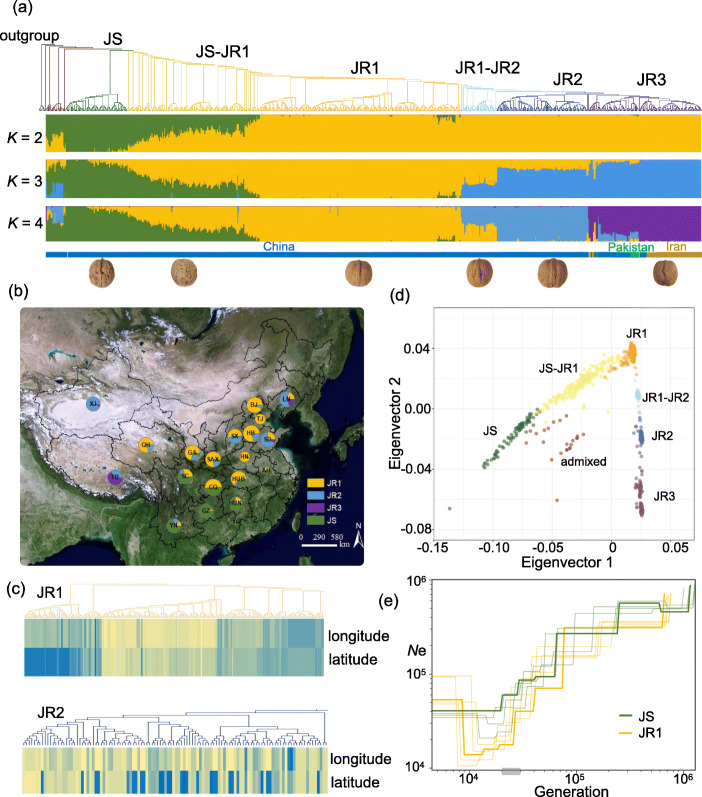
Population structure of *J. sigillata* and *J. regia* samples in this study. **a** Phylogenetic tree and population structure of *J. sigillata* and *J. regia* accessions. The order of accessions in the population structure plot is according to the phylogenetic relationship in the tree. One *J. sigillata* (JS) and three *J. regia* groups (JR1, JR2, and JR3) are clearly separated from each other and colored dark green, orange, blue, and purple, respectively. JS-JR1 and JR1-JR2 are admixed populations based on the ancestry inference. The outgroup clade including 8 *Juglans* accessions (2 accessions each for *J. cathayensis*, *J. mandshurica*, *J. microcarpa*, and *J. nigra*) is colored in black. The information for the country origins of the samples is indicated on the bottom. **b** Geographic distribution of the Chinese *Juglans* samples. The different colors in the pies represent ancestral components (according to the substructure at *K* = 4), while population size is indicated by the radius. **c** Different kinship patterns between the phylogenetic tree and geographic locations for JR1 and JR2. The colors are scaled by longitude or latitude values of each accession. **d** Principal component analysis (PCA) plot for 818 *J. regia* and *J. sigillata* accessions. **e** Effective population size (*N*e) changes for JS and JR1. The estimated divergence time (generation) between JS and JR1 is shown in the gray-shaded bar along the *x*-axis

To better infer the phylogenetic relationship and population structure of walnut, we integrated publicly available genomic data of 11 samples covering 6 *Juglans* species (one for *J. regia*, and two each for *J. sigillata*, *J. cathayensis*, *J. mandshurica*, *J. microcarpa*, and *J. nigra*) [3] (Additional file 1: Table S1). Based on the phylogenetic tree, we found that, *J. regia* (JR) and *J. sigillata* (JS) accessions could be separated into different clades, while there is one intermediate group between the two species clades (*K* = 2, Fig. 1a). The JS genetic composition for the samples of the intermediate group shows a progressively declining pattern shifting from JS to JR group. The genetic composition is generally consistent with their geographic distributions (Fig. 1b). From Southwest to North China, the JS samples are predominantly located in Southwest areas (Yunnan and Guizhou provinces), individuals of the intermediate type are distributed in Middle China (Sichuan and Chongqing provinces), while most JR samples are in provinces with relatively high latitudes. Notably, the *J. sigillata* group is located at the basal position in the phylogenetic tree close to the four outgroup *Juglans* species, possibly indicating that *J. sigillata* was ancestral to *J. regia*.

Based on the maximized marginal likelihood value estimated by fastSTRUCTURE [18], *K* = 4 was the optimal grouping number (Fig. 1a; Additional file 2: Fig. S2). At this *K* value, we found the JR accessions could be further divided into 3 main subgroups (JR1, JR2, and JR3), in which JR1 mainly consists of accessions from Middle and North China, JR2 accessions are mainly distributed in Northwest (Xinjiang) and Northeast (Liaoning) China, while the JR3 subgroup consists of accessions from Iran, Pakistan, and the Tibetan Plateau, China (Fig. 1a, b). We found a much higher proportion of improved *J. regia* varieties in the JR2 (39/114) than in JR1 (14/251), suggesting that JR2 seems to be subjected to more artificial selection and is likely a more derived group compared to JR1 (Additional file 1: Table S1). Consistently, JR2 also showed favorable traits including higher single nut weight, larger nut size, and higher fatty acid content compared to JR1 and JS (Additional file 2: Fig. S3,4). We further examined the relationship between the phylogenetic order and detailed geographical locations of each sample in JR1 and JR2. Interestingly, we found that in contrast to the JR1 population in which individuals located in close geographical regions are clustered in the same sub-clade, samples in the potentially improved population (JR2) are scattered in different clades (Fig. 1c), indicating that samples may be artificially dispersed or immigrated during the process of walnut improvement.

Principal component analysis (PCA) based on the genome-wide SNPs supported the population structure evidenced in the phylogenetic tree (Fig. 1d). The JS and JR1 samples are “connected” by the intermediate group (JS-JR1), consistent with the ancestry inference pattern, suggesting extensive hybridizations between JS and JR1 after their divergence. We inferred the dynamic changes in effective population size (*N*e) for *J. sigillata* (JS) and *J. regia* (JR1) populations and found the *N*e of the JR1 population rapidly decreased relative to JS around 20–30 k generations ago (Fig. 1e), indicating that *J. regia* may be derived from *J. sigillata* and suffered a genetic bottleneck around 200–300 k (~ 10 year per generation) years ago.

### Genetic diversity of different walnut populations

**Fig. 2:**
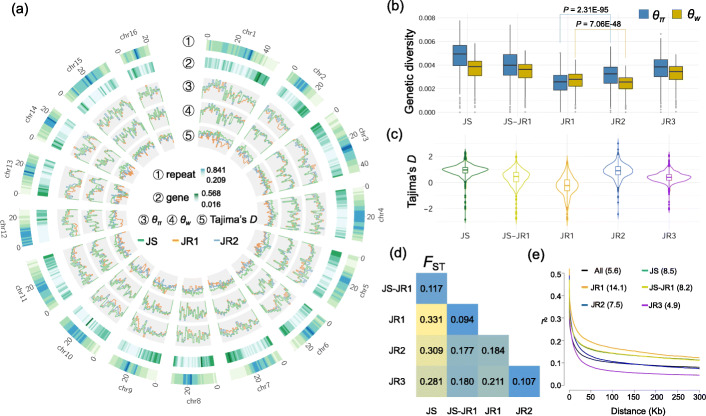
Genetic diversity and linkage disequilibrium for different walnut populations. **a** Circos plot for the genome-wide nucleotide diversity. Cycles from outside inwards: ① repeat density, ② gene density, the genome-wide distribution for *θ*_*π*_, *θ*_*w*_, and Tajima’s *D* values are shown in cycles ③, ④, and ⑤, respectively. The line colors for each population are indicated inside of the circos plot. For estimation of the gene or repeat density of each 1 Mb window, the lengths of genes or repeats within the window were summed up and then divided by the window length. **b** Boxplot of genetic diversity. The *θ*_*π*_ and *θ*_*w*_ values are indicated by dark blue and yellow, respectively. The *P* values are calculated based on pairwise *t-*test. **c** Distribution of Tajima’s *D* values. **d** Divergence of different walnut subpopulations estimated based on *F*_ST_. The summary statistics are calculated based on 1 Mb window size with a step size of 1 Mb. **e** Linkage disequilibrium differences between *J. sigillata* and various *J. regia* subpopulations

Two summary statistics, *θ*_*w*_ and *θ*_*π*_ values were calculated genome-wide to estimate the genetic diversity for different *Juglans* populations (Fig. 2a). Among the three Chinese *Juglans* populations (JS, JR1 and JR2), we found the JS population had obviously higher genetic diversity than the two Chinese *J. regia* populations (JR1 and JR2). The overall reduction in nucleotide diversity supports that Chinese *J. regia* could have endured a genetic bottleneck during divergence from *J. sigillata* (Fig. 2b; Fig. 1e). In addition, Tajima’s *D* values for JR1 were much lower compared to JS (Fig. 2c), which could be explained by fixation of alleles led by genetic drift. It is noteworthy that when comparing the diversity (*θ*_*w*_ and *θ*_*π*_) of JR1 and the improved JR2 population, we observed an opposite pattern based on the two different summary statistics (Fig. 2b). The JR1 population (2.648E−3) has a significantly higher genome-wide *θ*_*w*_ value than JR2 (2.428E−3) (*P* value = 7.06E−48, pairwise *t-*test). However, a significantly (*P* value = 2.31E−95, pairwise *t-*test) higher *θ*_*π*_ value was observed in the JR2 (3.113E−3) compared to JR1 (2.475E−3). The reduction in the *θ*_*w*_ value could be due to loss of segregation sites caused by genetic drift during the genetic bottleneck, while the heterogeneity across genome-wide loci was increased possibly due to artificial hybridizations during dispersal and immigration for improvement of walnut trees. We examined the diversity characteristics in some perennial tree species (Additional file 4: Table S3) and found that unlike common annual crops with dramatic reduction of genetic diversity during crop domestication and improvement, the genetic diversity of domesticated or improved perennial trees is not necessarily lower than their progenitors. For example, in the tea tree population studies [19, 20], the nucleotide diversity of cultivated tea plants is higher than their wild ancestors. In European pears, although the *θ*_*w*_ of wild population is much higher than domesticated one, the *θ*_*π*_ value, on the contrary, is slightly higher in the domesticated population [16] (Additional file 4: Table S3). A pairwise population differentiation (*F*_ST_) among the *Juglans* populations suggested that the inter-species divergence between *J. sigillata* and *J. regia* was around 0.28 to 0.33, while intra-species divergence within *J. regia* was 0.11–0.21 (Fig. 2d). We further evaluated the linkage disequilibrium (LD) for different walnut populations and found that the LD decay rate is rapid for walnut populations, with 5.6 kb (here measured as the distance at which the average pairwise SNP correlation coefficient *r*^*2*^ dropped to half of its maximum value) for the entire population (Fig. 2e). Consistent with the reduction in genetic diversity in the JR1 population, it had a much slower decay rate (14.1 kb) than JS (8.5 kb). A more rapid LD decay was observed in the Chinese improved *J. regia* group JR2. The genetic diversity of JR3 is higher than that of JR1 and JR2, and more rapid LD decay was observed in JR3. This could be due to that this population includes geographically widespread accessions, i.e., Iran, Pakistan, and the Tibetan Plateau, China.

### Characterization of deleterious mutations in walnut genomes

**Fig. 3:**
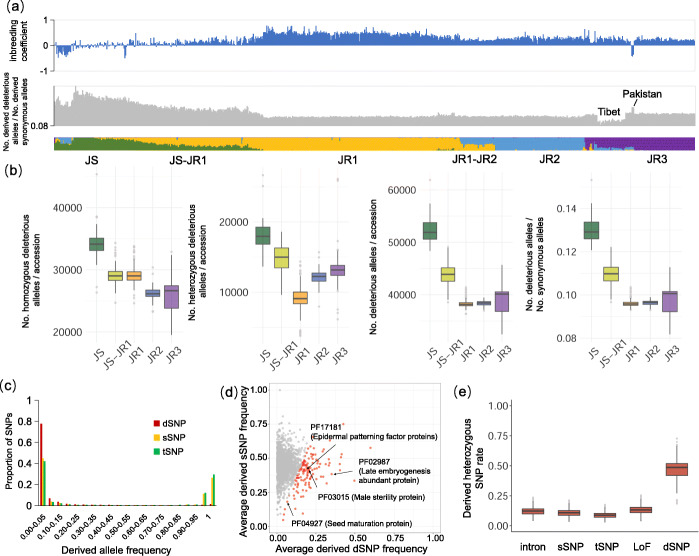
Deleterious allele landscapes in the genomes of different walnut population. **a** Top: heterozygous level for each walnut individual used in this study measured by the inbreeding coefficient. Middle: ratio of the number of derived deleterious alleles and the synonymous alleles of each sample. The samples from Tibet and Pakistan are labeled. Bottom: ancestry inference. The accession order is based on the phylogenetic tree in Fig. 1a. **b** The mutation burden in walnut populations. From left to right shows the total, homozygous, heterozygous, and normalized (dSNP/sSNP) mutation burden. **c** Site allele frequency of deleterious mutations in the walnut genome based on 818 samples. **d** The proportion of genes with normalized mutation burden (dSNP/sSNP). The proportion of genes with high mutation burden is highlighted in red. **e** The rate of derived heterozygous SNPs in genic regions for different walnut populations. “LoF” refers to “loss of function,” which is annotated as “high” effect SNP (e.g., stop-gain (nonsense), splice site-disrupting SNV) in the SNPeff software

The genetic burden has been examined in many annual crops such as rice, maize, and rapeseed, revealing accumulation of deleterious alleles due to “domestication cost” [21–24]. However, very few studies have investigated the change in genetic burden during the adaptation and improvement of perennial trees [25]. To evaluate the individual mutation burden for *J. sigillata* and *J. regia* samples, we used *J. nigra* as an outgroup to polarize the allele frequency. We found that the *J. sigillata* group generally has higher genomic heterozygosity than *J. regia*. Similarly, higher genetic burden was observed in *J. sigillata* than in *J. regia*, and the burden of the intermediate group (JS-JR1) between JS and JR1 decreased with the increased proportion of the JR1 ancestry (Fig. 3a). In addition, the collection of walnut samples from Pakistan (within JR3) harboring high genomic heterozygosity also had high genetic burden (Additional file 2: Fig. S5), indicating that heterozygous deleterious SNPs play a crucial role in the walnut evolution. Notably, the deleterious burden in the Tibetan walnut population is lowest (Additional file 2: Fig. S5), which could reflect a stronger purifying selection act on this walnut population living in a harsh environment for better fitness.

When calculating the homozygous and heterozygous deleterious alleles, we found that the proportion of heterozygous alleles for different *Juglans* populations ranged from 23.8 to 34.5%, which is higher than cultivated rice (< 5%) [21] but lower than clonally propagated plants like cassava (60–70%) [26] and grape (70–80%) [27]. The numbers of homo- and heterozygous deleterious alleles are higher in *J. sigillata* than in *J. regia* (Fig. 3b). During the improvement of *J. regia* (JR1 to JR2), although the total genetic burden remained generally unchanged, the proportion of heterozygous deleterious mutations for JR2 increased substantially compared to its progenitor JR1 (Fig. 3b).

We observed that the deleterious SNPs segregated at a lower frequency compared to tolerant and synonymous SNPs in walnut genomes, suggesting a stronger effect of purifying selection acting on the dSNPs than other types of SNPs during walnut evolution (Fig. 3c). We further investigated the putative functions of the genes with high genetic burden. The average derived dSNP and synonymous SNP (sSNP) frequency for genes of the same Pfam were calculated, and we found that genes with Pfams like PF02987 (Late embryogenesis abundant protein), PF03015 (Male sterility protein), PF04927 (Seed maturation protein), and PF17181 (Epidermal patterning factor proteins) are among groups of relatively higher deleterious allele fixation rate (top 5%) (Fig. 3d; Additional file 5: Table S4), indicating a high genetic load for these genes for walnut. Interestingly, we observed an elevated percentage of derived heterozygous dSNPs (46.9%) compared to sSNPs (10.9%) and even SNPs causing loss of functions (LoF) (13.3%). This may indicate that deleterious alleles are more likely to be masked in heterozygous form in walnut trees possibly as an evolutionary strategy for better adaptation (Fig. 3e).

### Signatures of selection in walnut genomes

**Fig. 4:**
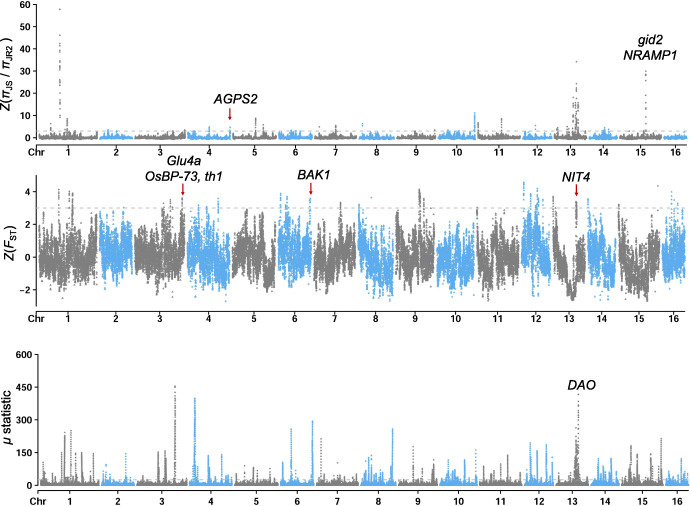
Genomic signatures for improvement of *J. regia*. Candidate genomic regions under positive selection possibly involved in improvement of *J. regia* (JR2). Selection signatures identified by three approaches are illustrated in the three subfigures, i.e., reduction of genetic diversity (top), genomic differentiation based on *Z*(*F*_ST_) (middle), and *μ* statistics (bottom). Four of the uniquely selected regions in JR2 are highlighted with red arrays

To identify candidate genomic regions with signatures of adaptation and improvement of *J. regia* populations (JR1 and JR2), we used *J. sigillata* as a background population and adopted a combination of three strategies (i.e., *Z* (*π*_JS_ / *π*_JR_) > 3, Z(*F*_ST_) > 3, and top 5% for *μ* statistic) to search for selective sweep regions (Fig. 4; Additional file 2: Fig. S6). In total, we identified 1961 and 2002 genes under putative selective regions for JR1 and JR2, respectively (Additional file 6: Table S5; Additional file 7: Table S6). Among the 2002 genes, there were 902 genes specifically selected in JR2, indicating they might be targets during walnut improvement. Gene ontology (GO) enrichment terms for genes selected in JR1 were like “pollen-pistil interaction” (GO:0009875), “recognition of pollen” (GO:0048544), and “response to biotic stimulus” (GO:0009607), suggesting that some selected genes in JR1 could be involved in species differentiation and environmental adaptation. For the uniquely selected genes in JR2, the GO categories “metal ion transport” (GO:0030001), and “lipid biosynthetic process” (GO:0008610) were overrepresented (Additional file 8: Table S7).

We found genes responsible for flowering time (e.g., *etr2*, *soc1*, *SPIN1*, and *LFY*) were located within the selective sweep regions, suggesting that those genes might play roles in adapting to high latitude environments for JR1 compared to JS (Fig. 1b; Additional file 2: Fig. S6). The tree height of *J. regia* is normally reduced compared to *J. sigillata* (Additional file 2: Fig. S7). Three genes (*gs1;1*, *gid2*, and *Slr1-d*) associated with dwarfism were also found in the target regions of JR1. Among them, we found the signature of positive selection of the region covering *gid2* was stronger in the JR2 (Fig. 4; Additional file 2: Fig. S6), indicating this region was under strong artificial selection during improvement. Interestingly, there existed a Mb-scale genomic region (Chr12: 1.3–14.1 Mb) which was highly differentiated (*Z*(*F*_ST_) > 3) between JR1 and JS. The region could also be targeted by signatures based on reduction of diversity and *μ* statistics. This genomic region included functional genes involved in flowering time (*LFY*, *VIM1*, and *TIC*) and reproduction (*gsl8-2* and *tms*) (Additional file 6: Table S5), which could be critical selection targets for *J. regia*.

Compared to JR1, we found that some genes located in regions specifically selected in JR2 are homologous to important functional genes responsible for seed weight and starch accumulation (Fig. 4; Additional file 7: Table S6). For example, a homolog of *BAK1* (JreChr06G11560) was found in the region (Chr6: 28.1–28.4 Mb) only differentiated between JR2 and JS. In rice, *BAK1* was reported to be involved in the developmental process of grain filling [28]. One homolog of *AGPS2* (JreChr04G11920) was in a genomic region (Chr4: 37.4–37.7 Mb) with specific reduction of diversity in JR2 compared to JS. *AGPS2* could be coordinated with starch accumulation in the developing rice seed [29]. Moreover, two *glu4a* genes (JreChr03G12610 and JreChr03G12612) involved in seed glutelin quality (eating quality) [30], and one *th1* gene (JreChr03G12627) related to seed weight per panicle in rice [31], were found within the candidate selected region (Chr3: 42.5–42.7 Mb). In addition to genes related to seed traits, we found two genes coding nitrilase with Pfam “PF00795” (carbon-nitrogen hydrolase) within a selected region (Chr13: 20.9–21.3 Mb) identified by two selective approaches (Additional file 7: Table S6). The two genes are homologous to *OsNIT1*, which has been reported to be involved in root-growth responses to nitrate and ammonium [32].

### Genomic variants associated with environmental factors

**Fig. 5:**
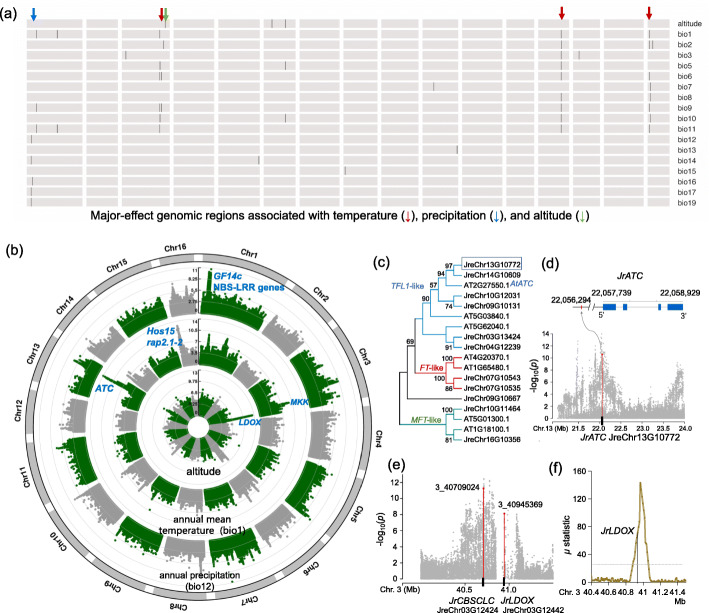
Genomic loci associated with environmental factors. **a** Summarized genomic loci associated with environmental factors related to temperature, precipitation, and altitude. The major-effect genomic loci associated with temperature, precipitation, and altitude are highlighted with red, blue, and green arrays, respectively. **b** Manhattan plots for annual mean temperature (bio1), annual precipitation (bio12), and altitude. **c** Phylogenetic tree for the genes of *J. regia* and *A. thaliana* in the phosphatidylethanolamine-binding protein (PEBP) gene family. The branches for subfamilies *TFL1-*like, *FT*-like, and *MFT*-like are colored in blue, red, and green, respectively. **d** A zoomed-in plot for the loci on chromosome 13 associated with temperature. One SNP located in the promoter region of *JrATC* is within the peak region. **e** A zoomed-in plot for the loci on chromosome 3 associated with altitude. **f** *JrLDOX* is located in the selected genomic region identified in Tibetan walnut population

By correlating environmental variables with genomic variants, it is possible to identify genomic loci that are involved in mediating adaptation. We focused on 20 environmental variables related to temperature, precipitation, and altitude, which are principal determinants of plant distribution and survival (Additional file 9: Table S8; Additional file 2: Fig. S8). A total of 48 genomic loci were identified which were likely related to the 20 environmental variables (temperature related: bio1-bio11, precipitation related: bio12-bio19, and altitude) (Fig. 5a; Additional file 10: Table S9; Additional file 11: Table S10). Three and one genomic hotspot regions were associated with temperature and precipitation, respectively, according to the results of multiple (> 3) genetic-environmental association (GEA) studies (Fig. 5a, b). For the three temperature-associated genomic hotspots on chromosomes 3, 13, and 16, we found genes homologous to functional genes responsible for cold tolerance in rice (*OsMKK6*) and in *Arabidopsis* (*hos15*, *rap2.1-2*) (Fig. 5b; Additional file 11: Table S10). Notably, one *J. regia* homolog (JreChr13G10772) of the *ATC* in the *Arabidopsis TERMINAL FLOWER 1* (*TFL1*) gene family was also located within the peak region on chromosome 13 (Fig. 5b–d). In plants, the *TFL1* gene family has been found to not only be involved in repression of flowering under short photoperiods, but can also regulate development in response to temperature [33].

For the hotspot region associated with precipitation (Fig. 5a, b), we found a gene cluster coding 13 nucleotide-binding site leucine-rich repeat (NBS-LRR) disease-resistant proteins. The enrichment of the NBS-LRR gene was also observed in a GEA study for peach adaptation [34]. In addition, SNPs within one gene (JreChr01G13585) with Pfam “PF02893” (GRAM domain) were significantly associated with precipitation variables. The GRAM gene family is part of the ABA signaling pathway [35]. In rice, a GRAM domain-containing protein has been reported to confer drought and salt tolerance [36].

UV-B radiation stress in high-altitude regions can enhance the production of reactive oxygen species (ROS), which could cause damage to plant growth and development. It has been documented that flavonoids, especially flavonols, are highly effective in mitigating ROS and UV-B radiation [37]. Interestingly, among the genomic region (on Chr3) most associated with altitude (Fig. 5b, e), we found one gene (JreChr03G12442) coding leucoanthocyanidin dioxygenase (*LDOX*) involved in the flavonoid biosynthetic pathway. In addition, the gene was also under positive selection in the walnut population collected from Tibet at high altitude (Fig. 5f; Additional file 2: Fig. S9; Additional file 12: Table S11). In addition, one *CBSCLC* gene (JreChr03G12424) with Pfam “PF00654” (Voltage gated chloride channel) is located within the peak region on Chr3. The *CBSCLC* gene family has been reported in response to various plant stresses including salinity, drought, cold, high temperature, UV, wounding, and genotoxicity, in both root and shoot tissues [38]. These genes warrant attention in future research to evaluate whether and how they are involved in mediating adaptation to climate in walnut species.

### Genome-wide associations with agronomic traits

A total of 44 traits were measured, which include phenotypic categories like yield components, fatty acid composition, and coloration (Additional file 12: Table S12; Additional file 2: Fig. S3). We then performed GWAS for these traits investigated in 2016 using common SNPs with minor allele frequency above 0.05. With a threshold of *P* < 1.16E−6, a total of 27 genomic loci were identified by both fastGWA and EMMAX software as significant GWAS peaks for 18 traits (Additional file 2: Fig. S10; Additional file 14: Table S13; Additional file 15: Table S14). For the 14 traits investigated in 2019, 17 associations underlying 7 traits were overlapped with those identified by our first GWAS analyses, with traits like leaflet and nut shape, number of single fruit, color of endopleura, and nut shell thickness showing good consistency of association signatures (Additional file 16: Table S15). The lead SNPs within the peak regions could be designed as markers and utilized by walnut breeders to select individuals with desirable traits (Additional file 15: Table S14).

**Fig. 6:**
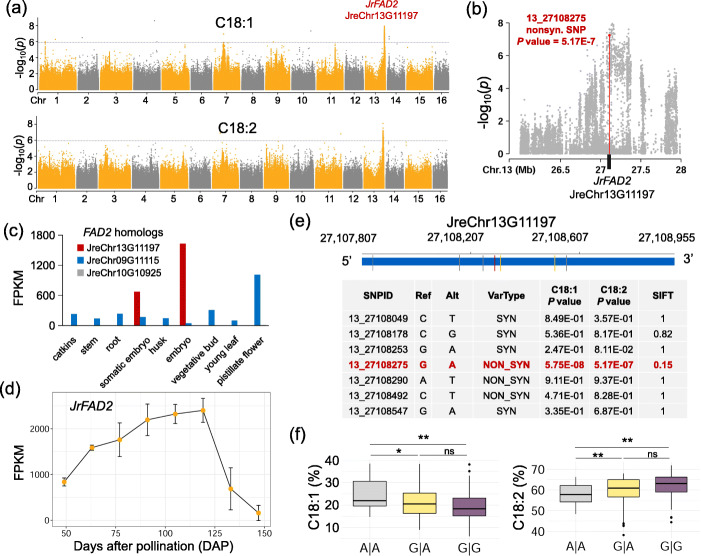
Candidate causative genes and variants for C18:1 and C18:2. **a** Manhattan plots of GWAS for C18:1 and C18:2. The genome-wide significant value threshold (1.16E−6) is indicated by a horizontal dash–dot line. One gene (JreChr13G11197) homologous to *FAD2* of *A. thaliana* resides in the GWAS peak region. **b** A zoomed-in plot for the GWAS peak of C18:2. One significantly associated nonsynonymous SNP (*P* value 5.17E−7) of *FAD2* is highlighted. **c** Expression level of three *FAD2* homologs. The JreChr13G11197 with significantly associated nonsynonymous SNP has the highest expression level in the embryo compared to other tissues. **d** Expression levels of JreChr13G11197 (*JrFAD2*) at different days after pollination (DAP). Three replicates for each time point. **e** Conservation level of the candidate causal SNP associated with C18:1 and C18:2 content. Top: locations of SNPs in the CDS region of JreChr13G11197; bottom: *P* values and conservation scores for different SNPs. The conversation score is measured based on the SIFT score (with lower values indicating higher conservation). **f** Differences in C18:1 and C18:2 contents with different alleles of the nonsynonymous SNP (13_27108275). *P < 0.05, **P < 0.01, Student’s *t* test

The ratio of unsaturated fatty acid relative to saturated fatty acids is an important indicator of evaluating the quality of edible oil. Compared to a stable unsaturated fatty acid proportion in sesame [39], a highly variable range of linoleic acid (C18:2) content (38.2–71.9%) was observed in walnut. GWAS was performed for the contents of oleic acid (C18:1) and linoleic acid (C18:2), and both traits were found to be associated with SNPs in one genomic region ranging from 27.0 to 27.5 Mb on chromosome 13 (Fig. 6a). Notably, one *FAD2* homologous gene (JreChr13G11197) was found within this region, of which one nonsynonymous SNP (13_27108275) showed a significant association with both C18:1 (5.75E−8) and C18:2 (5.17E−7) (Fig. 6b; Additional file 15: Table S14). *FAD2* encodes an oleic acid desaturase, which converts oleic acid to linoleic acid in the endoplasmic reticulum [40]. There are three paralogous genes encoding *FAD2* in the walnut genome, among which the candidate gene JreChr13G11197 was found to be highly expressed in the embryo and somatic embryo compared to other paralogs (Fig. 6c). In our earlier study [41], the biosynthesis efficiency of linoleic acid was found to be highest between 91 and 119 days after pollination (DAP). We examined the transcriptomic profiles of JreChr13G11197 at different time points after pollination, and observed similar pattern between its expression level and the efficiency of linoleic acid biosynthesis (i.e., highest expression between 91 and 119 DAP) (Fig. 6d), adding more transcriptomic evidence that the JreChr15G11197 (*JrFAD2*) is the causal gene responsible for linoleic acid biosynthesis in walnut. We further evaluated the conservation level based on SIFT (sorting intolerant from tolerant) score [42] (Fig. 6e) and found the significantly associated nonsynonymous SNP (13_27108275) was also of higher conservation than other variants within the gene, indicating that it might be a causative SNP responsible for the trait. As expected, samples harboring the GG allele had significantly lower oleic acid but higher linoleic acid content than those with the GA and AA alleles (Fig. 6f).

**Fig. 7:**
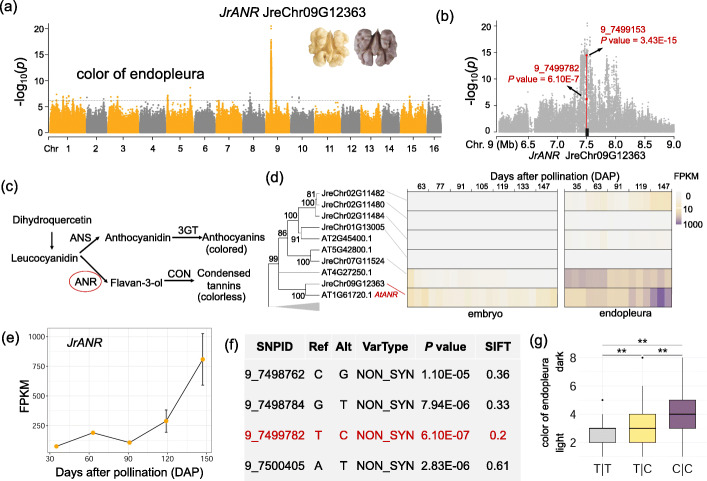
Candidate causative gene for walnut endopleura color. **a** Manhattan plot of GWAS for color of walnut endopleura. The homolog of *ANR* (JreChr09G12363) located in this region is highlighted. Walnut fruits with contrasting bright and dark colors are shown. **b** Zoomed-in plot for the GWAS peak associated with endopleura color. SNPs in theA NR gene are highlighted. **c** Relationship between the biosynthesis of condensed tannins and anthocyanins. **d** Identification of ortholog of *AtANR* in walnut (left). Expression profiles of *ANR* homologs at different developmental stages of embryo and endopleura in walnut (right). Three replicates for each time point. **e** Expression levels of JreChr09G12363 (*JrANR*) at different days after pollination. **f** Conservation level (based on the SIFT score) of nonsynonymous SNPs in the *ANR* gene. **g** Comparison of walnut endopleura color for samples with different alleles for the potentially causative SNP (9_7499782). **P < 0.01, Student’s *t* test

The color of endopleura is one of the most commercially important characteristics of walnut. There exists one major loci on chromosome 9 significantly associated with the color trait (Fig. 7a; Additional file 15: Table S14). Zooming in on the GWAS peak region, we found a gene JreChr09G12363 homologous to *ANR* (*BAN*) located within this region (Fig. 7b). *ANR* encodes an anthocyanidin reductase, which functions as a negative regulator of flavonoid biosynthesis and is involved in procyanidin accumulation in the seed (Fig. 7c) [43]. We further examined the transcriptomic profile of this gene in embryo and endopleura (Fig. 7d) and found a stable and moderate expression level of this gene in embryo during different time points. However, during the development of endopleura, the expression level of JreChr09G12363 (*JrANR*) showed a continuously increasing trend, and FPKM value could reach up to ~ 800, providing more support for a role of *JrANR* playing in the endopleura development (Fig. 7d,e). Within the gene, one nonsynonymous SNP (9_7499782, T to C) showed a significant signal (*P* = 6.1E−7) (Fig. 7f). Moreover, this missense SNP showed the highest conservation level compared to the other nonsynonymous SNPs in the gene (Fig. 7f). Samples with the “CC” allele of this SNP showed significantly darker endopleura color than those with the “CT” and “TT” alleles (Fig. 6g). Nut shell thickness has been regarded as a crucial commercial trait for walnut, with thinner shell being easier to crack (Additional file 2: Fig. S11). For this trait, we also identified one associated genomic peak on chromosome 5, which includes one nonsynonymous SNP for *MYB16* (JreChr05G10191_01) (Additional file 15: Table S14). The homolog gene (AT5G15310) in *Arabidopsis* has been proved to promote unidirectional cell expansion and can participate in the regulation of cuticle biosynthesis and wax accumulation in reproductive organs and trichomes [44]. These identified genes related to the economically important agronomic traits can be potentially used for the improvement of walnut trees.

## Discussion

In genetic studies of annual crops, plants are usually collected and grown in the same environments for investigation of their fitness or agronomic traits during their 1-year life span. For long-lived crop trees with a long juvenile period (e.g., 7 ~ 15 years for common walnut), using a similar strategy would involve a tremendous amount of time and efforts to investigate their flowering time and fruit traits. Considering the differences between long-lived perennial trees and annual crops, strategies to study their genetic basis of adaptation and agronomic traits may be modified accordingly. It would be more feasible to first identify one genetically diverse tree population and investigate the phenotypes of the same individuals in the population annually. Here, we constructed a genotyping panel of 815 walnut trees, among which annually continuous phenotyping could be performed for walnut individuals from China. This phenotyping strategy in a tree population has been proved to be capable of mapping candidate QTLs for some important traits investigated in this study. With accumulated annual phenotyping data, more GWAS analyses and evidences could refine these QTLs. This may also assist in determining the genetic basis of traits related to tree development. In addition to agronomic traits investigated here, many other traits like disease resistance, root morphology, and even microscopic phenotypes of walnut trees could also be studied based on this population and genotyping panel in the future.

Marker-assisted selection (MAS) and genome selection (GS) have been successfully applied to accelerating breeding cycles for many annual crops. Due to the long breeding period of fruit trees, it is important to develop useful MAS and GS tools for the selection of individuals that have desirable traits and good fitness to the growing environments [45]. Based on the GWAS and GEA analyses for walnut population in this study, we noticed that agronomic traits like color of endopleura and linoleic acid content are likely to be controlled by 1 or 2 major-effect quantitative trait loci (QTLs). Moreover, luckily, the adaptations of walnuts to stresses related to temperature, precipitation, and altitude also seem to be controlled by major-effect QTLs. For these traits, a library of markers could be constructed based on lead or even causative variants, which could help speed up the selection of desired individuals in an effective and cost-efficient manner [46]. For traits controlled by minor-effect QTLs, GS models could be developed taking advantage of the walnut variation map constructed in this study.

Although genomics has greatly accelerated the dissection of potential genes or networks related to fruit tree traits in the past decade, there remain many limitations which hinder further causative gene identification and in-depth gene function verification. For example, it is usually difficult to construct an artificial population for a perennial tree species. The lack of knowledge on gene function in fruit trees also presents an obstacle to narrow down candidate genes in the GWAS analysis. Functional analysis requires simple and effective transgenic technologies. While a mature transformation approach in walnuts (and other fruit trees) was developed some time ago [47], it is still laborious to study the gene function in vivo due to the long-life cycle. Furthermore, it is also not appropriate to verify some fruit-related traits ectopically in annual model plants. Therefore, there is still much scope for future functional genomics research in crop trees.

## Conclusions

Based on the most comprehensive walnut genomic variation map to date, we found that Chinese *J. regia* likely diverged from *J. sigillata* with a genetic bottleneck. Extensive hybridizations occurred after the divergence of the two species. The improvement in the walnut trees was accompanied with an increased level of heterozygosity and genetic diversity, which is distinct from the cultivation process of annual crops. The deleterious mutations in walnut tree genomes were shifted from a homozygous to a heterozygous form during its improvement. The signatures of environmental adaptation and improvement for walnut trees were uncovered by selective sweep scans and genetic-environmental associations. We also identified potentially causative genes for economically important traits including linoleic acid content and color of endopleura. These identified loci will contribute significantly to the genetic improvement and molecular breeding of walnuts.

## Methods

### Plant sampling and DNA sequencing

We collected 815 walnut samples from China (728), Iran (76), and Pakistan (11). The Chinese walnut accessions were widely sampled from 19 provinces (Fig. 1a). Paired-end sequence data were generated by Illumina NovaSeq. The average sequencing depth for the generated genomic data is 19.0×. Among the 728 walnut samples collected in China, 92 accessions were approved as improved varieties by walnut variety approval committees from each province in China. The approved walnut varieties are normally characterized by high yield (e.g., high fruit and nut weight), favorable fruit quality (e.g., light colored kernel, tasty nut), and good resistance to pathogens and are able to adapt well to local agro-ecological conditions. In addition, to facilitate inference of the phylogeny of these samples, we also integrated 11 publicly available genomic datasets concerning *Juglans* accessions from Zhang et al. (2019) [3], including *J. sigillata* (2), *J. regia* (1), *J. cathayensis* (2), *J. mandshurica* (2), *J. microcarpa* (2), and *J. nigra* (2) (Additional file 1: Table S1).

### Phenotyping

A total of 44 agronomic traits of walnut accessions collected from China were investigated in 2016 (44 traits) and in 2019 (14 traits) (Additional file 13: Table S12), including yield formation, nut and leaf characteristics, tree and biological characteristics, and quality characteristics. Nut-related traits were measured using fully matured nuts in the laboratory. Vertical diameter, transverse diameter, lateral diameter, nut shell thickness, and hull thickness were measured using a digital caliper or spiral micrometer (Additional file 17: Supplementary Note). Protein content was measured with the Kjeltec 8400 Analyzer (Foss, Sweden) according to the user manual. The fatty acid composition was determined by a 7890A gas chromatogram (Agilent Technologies) according to a previous report [48]. Walnut endopleura colors were categorized into different levels from light to dark. The following phenotypes were determined by visual observation: shell surface feature, nut shape, nut top shape, nut shoulder shape, nut bottom shape, suture feature, nut inner wall, nut diaphragm, kernel plumpness, nut uniformity, tree posture, number of side shoots withdrawing fruit, crown shape, leaflet shape, parietal degeneration, leaf tip shape, and leaf edge shape (Additional file 17: Supplementary Note).

### Variant calling and genotyping

Sequencing reads of each walnut accession were mapped to *J. regia* genome [9] using BOWTIE2 v2.2.1 [49] with default settings. Consecutive steps using Samtools v0.1.19 [50] and GATK v3.7 [51] were applied for variant detection. Potential PCR duplicates were removed using “Samtools rmdup.” Alignments around small indels were realigned with “IndelRealigner,” and raw variants were called based on the realigned bam file. The resulting BAM file for each walnut sample was used for the multi-sample variant genotyping. “UnifiedGenotyper” in GATK v3.7 was applied to generate the raw variant calls with parameters “-stand_call_conf 30, -stand_emit_conf 10.” To reduce the variants false discovery rate, the variant calls were further filtered according to the following threshold: QUAL < 30, QD < 2, MQ < 30, and MQ0/DP > 0.1. Potential variant annotation and effect were predicted by SnpEff v3.6 [52]. To evaluate the SNPs genotyping accuracy, we performed Sanger sequencing for six genomic segments for 12 to 19 walnut accessions. The primers for each segment are listed in Additional file 18: Table S16.

### Population structure and LD analysis

A phylogenetic tree was constructed using FastTree [53] based on the 571,589 SNPs at four-fold degenerate (4DTv) sites among 815 walnut accessions and 11 *Juglans* individuals from publicly available data. Among the 11 *Juglans* individuals, 8 accessions including *J. cathayensis*, *J. mandshurica*, *J. microcarpa*, and *J. nigra* were used as the outgroup. Principal component analysis was performed for 818 *J. regia* and *J. sigillata* samples by SNPRelate v0.9.19 [54]. The ancestry inference was undertaken using FastStructure [18] for the 818 accessions with *K* values ranging from 2 to 10. The module implemented in fastSTRUCTURE “chooseK.py” was used to identify the proper *K* value that maximized the marginal likelihood. Two summary statistics *θ*_***w***_ and *θ*_***n***_ values for diversity estimation for each population were calculated by VariScan [55] based on a sliding window size of 1 Mb. LD decay statistics were calculated for different populations by PopLDdecay3.26 (https://github.com/BGI-shenzhen/PopLDdecay). SNPs with minor allele frequency (MAF) above 0.05 were used for LD estimation of each population. We estimated the inbreeding coefficient for each individual of all walnut samples by VCFtools (v0.1.13, https://vcftools.github.io) [56]. The software estimates the inbreeding coefficient for each individual in the genotyping VCF matrix based on the equation *F* = (*O*_(hom)_ − *E*_(hom)_) / (*N* − *E*_(hom)_), in which *O*_(hom)_ is the observed number of homozygous SNPs, *E*_(hom)_ refers to the expected number of homozygous SNPs, and *N* is the total number of genotyped loci of the individual.

### Demography inference

The divergence time between *J. sigillata* and *J. regia* was inferred using SMC++ v1.13 [57]. For each population of JS and JR1, we down-sampled the individual number to 40 (5 times) to improve the estimation accuracy of the effective population size. The divergence time range was estimated according to the distinct change of effective size between JS and JR1 populations. The mutation rate was assumed as *μ* = 2.5 × 10^−9^ mutations×bp^−1^ × generation^−1^ [58].

### Genome-wide selective sweep analysis

The genome was scanned in a 100-kb window size with a step size of 10 kb, and the population parameters (*π*, *F*_ST_) were estimated for each window by VCFtools (v0.1.13, https://vcftools.github.io) [56]. Using the *J. sigillata* population as background, the *F*_ST_ and relative genetic diversity change for the two *J. regia* populations (JR1 and JR2) were calculated and then were standardized and transformed into *Z*-scores. The genomic windows with a *Z*-score above 3 were determined as candidate regions. In addition, we used RAiSD [59] to detect candidate genomic regions under positive selection for *J. regia* populations (JR1 and JR2). The scanning window size of RAiSD was set to 10 kb. RAiSD adopted a composite evaluation test that scores genomic regions by quantifying changes in the amount of genetic diversity, SFS, and the levels of LD and along a chromosome [59]. The genes within the selective sweep regions identified by the three methods were taken as candidate genes under selection.

### SNP conservation and analysis of deleterious variation

For measuring SNP conservation, we used SIFT software [42] to construct a SIFT score database based on the *J. regia* reference genome. The SIFT score ranges from 0 to 1, with 0 being more conservative. For genes within GWAS significant signals, SIFT scores for SNPs at coding regions could be obtained.

For the deleterious variation analysis, we followed previous deleterious variation studies in plants (rice [21], grape [22], cassava [26]) and determined the deleteriousness of a SNP mutation site by assessing the degree of sequence conservation (based on the SIFT software [42]). The detailed steps to identify and calculate the number of homozygous and heterozygous deleterious alleles are as follows: (1) We first generated a combined SNP genotyping matrix including all *J. regia* and *J. sigillata* accessions as well as one *J. nigra* sample (outgroup). (2) The software SnpEff was applied to determine nonsynonymous SNP sites. We further used the SIFT software to calculate the SIFT score, which could estimate the probability of each nonsynonymous SNP to be deleterious (SIFT score < 0.05) or tolerant (SIFT score ≥ 0.05). (3) Since we aimed to calculate the derived deleterious allele for each sample, the genotypes of the *J. nigra* accession (homozygous state) was used for allele polarization. For a deleterious SNP site, if the SNP genotype of a given sample is homozygous and different from *J. nigra*, we determined it as homozygous deleterious SNP, then the number of derived deleterious alleles is 2 × No. homozygous deleterious SNPs. If the SNP genotype of a given sample is heterozygous, we determined it as heterozygous deleterious SNP, then the number of derived heterozygous deleterious alleles is 1 × No. heterozygous deleterious SNPs. Finally, the total number of deleterious alleles for each sample is 2 × No. homozygous derived SNPs + 1 × No. heterozygous deleterious SNPs.

### Identification of genetic variants associated with walnut traits and environmental data

Genome-wide association study was performed for 44 traits by fastGWA [60] implemented in GCTA [61] with the linear mixed model. PCA was controlled as fixed effect. To confirm the signatures identified by fastGWA, we also applied EMMAX software [62] to perform GWAS for all the phenotyped traits using linear mixed model. In our walnut collection, the total number of individuals with any phenotypic data is 612; therefore, 6,708,149 SNPs (MAF > 0.05) for the 612 accessions with phenotypes were used. We set the genome-wide significance of all traits with a uniform threshold (*P* = 1 / the effective number of independent SNPs) [63]. The independent number of SNPs (863,148) was determined by PLINK with the parameters “window size = 50, step size = 5, and *r*^2^ ≥ 0.5” [64]. Therefore, we set the GWAS threshold as 1.16E−6 (1 / 863,148).

For genetic-environmental association analysis, the latitude, longitude, and altitude data for each individual were recorded with a GPS measuring instrument (China, YiLi × 28). Based on the latitude and longitude of each walnut sample, we extracted 19 bioclimatic variables (bio1 – bio19) related to temperature and precipitation from the WorldClim 2 database [65] using the RASTER R package [66] (Additional file 9: Table S8). Since some collected Chinese *J. regia* accessions (mainly in JR2) could have migrated from different places, we only explore genetic-environmental associations based on samples from populations of JS, JS-JR1 and JR1. Taking environmental data as phenotypes, genetic-environment associations were performed using mixed linear model by fastGWA [60] with PCA controlled as fixed effects. Manhattan plots in the GWAS and GEA analyses were visualized using CMplot package [67].

### Gene expression profiling

The RNA-seq data for walnut embryo were from our previous study (Additional file 19: Table S17) [41]. Transcriptomic data for walnut endopleura samples were generated in this study. The samples were harvested at 35, 63, 91, 119, and 147 DAP during the walnut development stage from walnut cultivar “Linzaoxiang” at Chinese Academy of Forestry. At each time point, endopleuras collected from 7 nuts were mixed into one biological replicate, with three replicates at each stage. All of the samples were quickly frozen into the liquid nitrogen and stored at − 80 °C refrigerator for transcriptome sequencing. Total RNA was extracted using the universal total RNA kit (Tiangen, Beijing, China). Transcriptomic sequencing was performed on the NovaSeq 6000 platform at Novogene Company (Beijing, China). The reads were aligned to our generated *J. regia* genome reference [9] by Hisat2 v2.1.0 [68], and StringTie v1.3.5 [69] was adopted for quantification of expression.

### GO enrichment analysis

GO enrichment analysis was carried out using AgriGO (http://bioinfo.cau.edu.cn/agriGO/) [70]. The GO annotation was extracted from the *J. regia *annotation based on Interproscan v5 [71]. The *P* value criterion of < 0.05 was used for the considered enrichment GO terms.

## Supplementary Information

**Additional file 1: Table S1.** Data information for all walnut accessions used in this study.

**Additional file 2: Fig. S1.** Circos plot for the genome-wide SNP density. **Fig. S2.** Population structure of *J. sigillata* and *J. regia* accessions. **Fig. S3.** Clustering of 44 traits for walnut samples investigated in this study. **Fig. S4.** Comparison of traits related to walnut fruit weight, size and fatty acid content for different populations. **Fig. S5.** Mutation burden for different walnut subpopulations in the JR3 group. **Fig. S6.** Genomic signatures for selection of JR1 walnut population. **Fig. S7.** Phenotypic differences between *J. sigillata* (JS) and *J. regia* (JR). **Fig. S8.** Clustering of 20 environmental values for walnut samples investigated in this study. **Fig. S9.** Genomic signatures for the Tibetan walnut population. **Fig. S10.** Genomic loci associated with agronomic traits of walnut identified by GWAS. **Fig. S11.** Candidate gene associated with nut shell thickness.

**Additional file 3: Table S2.** Comparison of called genotypes by NGS-based bioinformatic approach and Sanger-based method.

**Additional file 4: Table S3.** Comparison of characteristics of genetic diversity during domestication and improvement of perennial trees.

**Additional file 5: Table S4.** Putative functions of genes with high genetic burden during walnut evolution.

**Additional file 6: Table S5.** Genes under putatively selected genomic regions for JR1 identified by three selective sweep scan approaches.

**Additional file 7: Table S6.** Genes under putatively selected genomic regions for JR2 identified by three selective sweep scan approaches.

**Additional file 8: Table S7.** Enriched GO terms for genes located in selected regions of JR1 and JR2.

**Additional file 9: Table S8.** Environmental data values for 492 walnut samples used for genetic-environment association analysis.

**Additional file 10: Table S9.** Genomic regions associated with environmental factors related to temperature, precipitation, and altitude.

**Additional file 11: Table S10.** Genes located in genomic regions identified by genetic-environmental association studies.

**Additional file 12: Table S11.** Genes under putatively selected genomic regions for the Tibetan walnut population identified by three selective sweep scan approaches.

**Additional file 13: Table S12.** Phenotypic values for walnut samples collected and investigated in 2016 and in 2019.

**Additional file 14: Table S13.** Genomic loci associated with agronomic traits investigated in 2016 in walnut identified by GWAS.

**Additional file 15: Table S14.** List of significantly associated genic SNPs (identified by fastGWA) for traits investigated in 2016.

**Additional file 16: Table S15.** Genomic loci associated with agronomic traits investigated in 2019 in walnut identified by GWAS.

**Additional file 17: Supplementary Note.** Detailed descriptions for the phenotyped walnut traits.

**Additional file 18: Table S16.** Designed primers for validation of called SNP genotypes.

**Additional file 19: Table S17.** Summary of RNAseq data used in this study.

**Additional file 20.** Review history.

## Data Availability

Short-read genomic resequencing data of 815 walnut samples generated by this study have been deposited into GenBank under the bioproject accession number PRJNA721107 (SRR14429941 - SRR14430755) [72]. The genomic data for 11 *Juglans* accessions from Zhang et al. can be downloaded from NCBI with bioproject accession PRJNA356989 [3]. The detailed sequence read archive IDs of genomic data are listed in Additional file 1: Table S1. The transcriptomic data for walnut endopleura samples have been deposited under bioproject accession PRJNA643637 (SRR15651947 - SRR15651961) and available in the NCBI Gene Expression Omnibus (GEO) repository accession GSE185230 [73]. The raw RNA-seq data for walnut embryo samples generated in our previous study are available with the bioproject accession PRJNA643637 (SRR12131480 - SRR12131503) [41]. The RNA-seq data of 9 different walnut tissues generated in our previous *J. regia* genome assembly study can be downloaded from PRJNA721107 (SRR15651918 - SRR15651926) [9]. The detailed sequence read archive IDs of transcriptomic data are listed in Additional file 19: Table S17. The SNP dataset has been deposited into the Genome Variation Map (https://ngdc.cncb.ac.cn/gvm/) under the accession number GVM000177. The codes for the bioinformatics analyses are provided open in the GitHub repository (https://github.com/lefroyqiu/walnut_pop_genomics_project) under MIT license [74]. The current release has also been deposited at the Zenodo repository (10.5281/zenodo.5544174) [75].
