# Supplementary material for: A genome variation map provides insights into the genetics of walnut adaptation and agronomic traits
Source: Genome Biol. 2021 Oct 27;22:300. doi: 10.1186/s13059-021-02517-6 (PMC8554829; doi:10.1186/s13059-021-02517-6)
Supplement: Supplementary file 2 — Additional file 2: Fig. S1. Circos plot for the genome-wide SNP density. Fig. S2. Population structure of J. sigillata and J. regia accessions. Fig. S3. Clustering of 44 traits for walnut samples investigated in this study. Fig. S4. Comparison of traits related to walnut fruit weight, size and fatty acid content for different populations. Fig. S5. Mutation burden for different walnut subpopulations in the JR3 group. Fig. S6. Genomic signatures for selection of JR1 walnut population. Fig. S7. Phenotypic differences between J. sigillata (JS) and J. regia (JR). Fig. S8. Clustering of 20 environmental values for walnut samples investigated in this study. Fig. S9. Genomic signatures for the Tibetan walnut population. Fig. S10. Genomic loci associated with agronomic traits of walnut identified by GWAS. Fig. S11. Candidate gene associated with nut shell thickness. [file 13059_2021_2517_MOESM2_ESM.pdf]

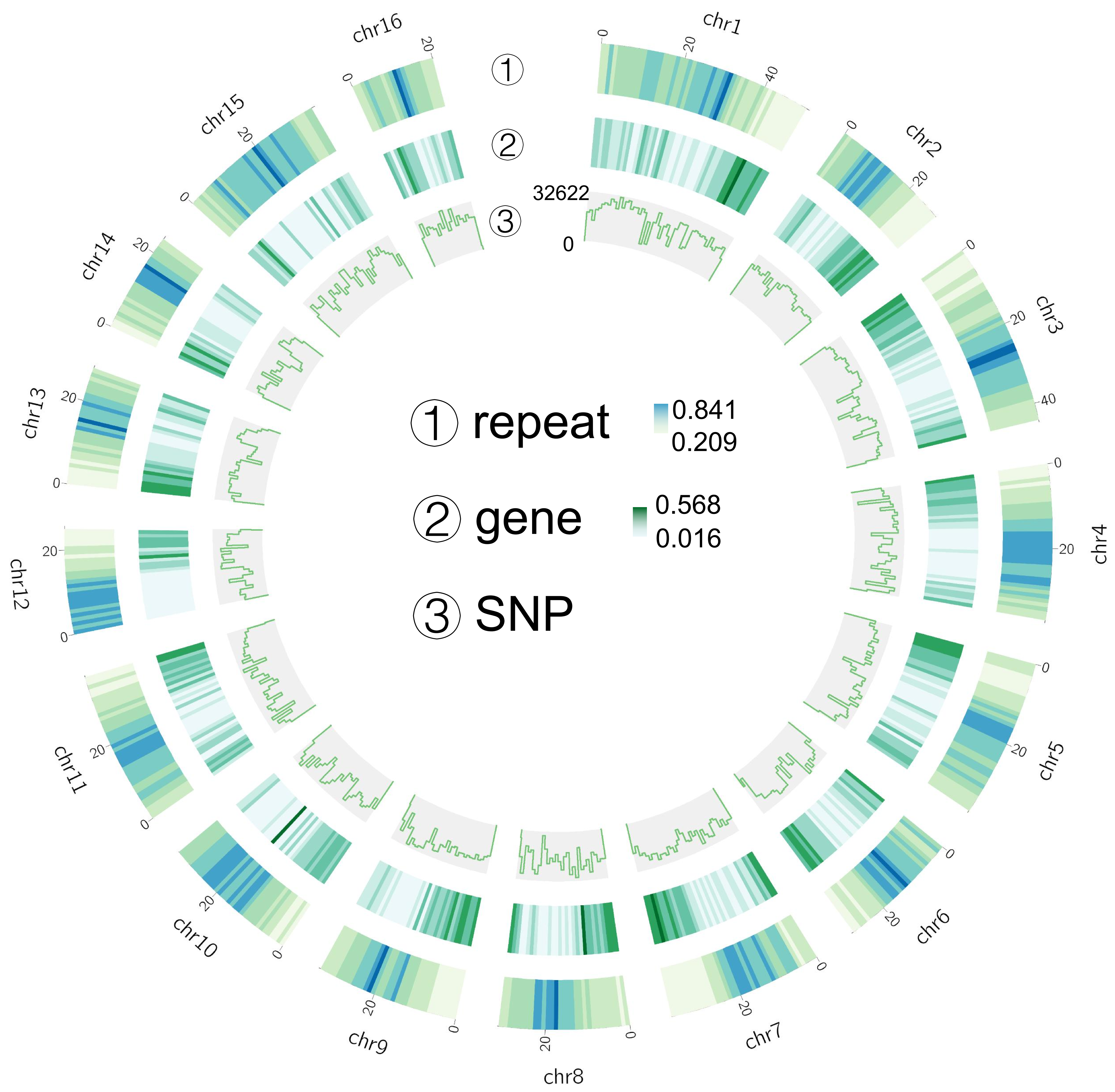

Fig. S1 | Circos plot for the genome-wide SNP density. Cycles from outside inwards: ① repeat density, ② gene density, ③ SNP density.

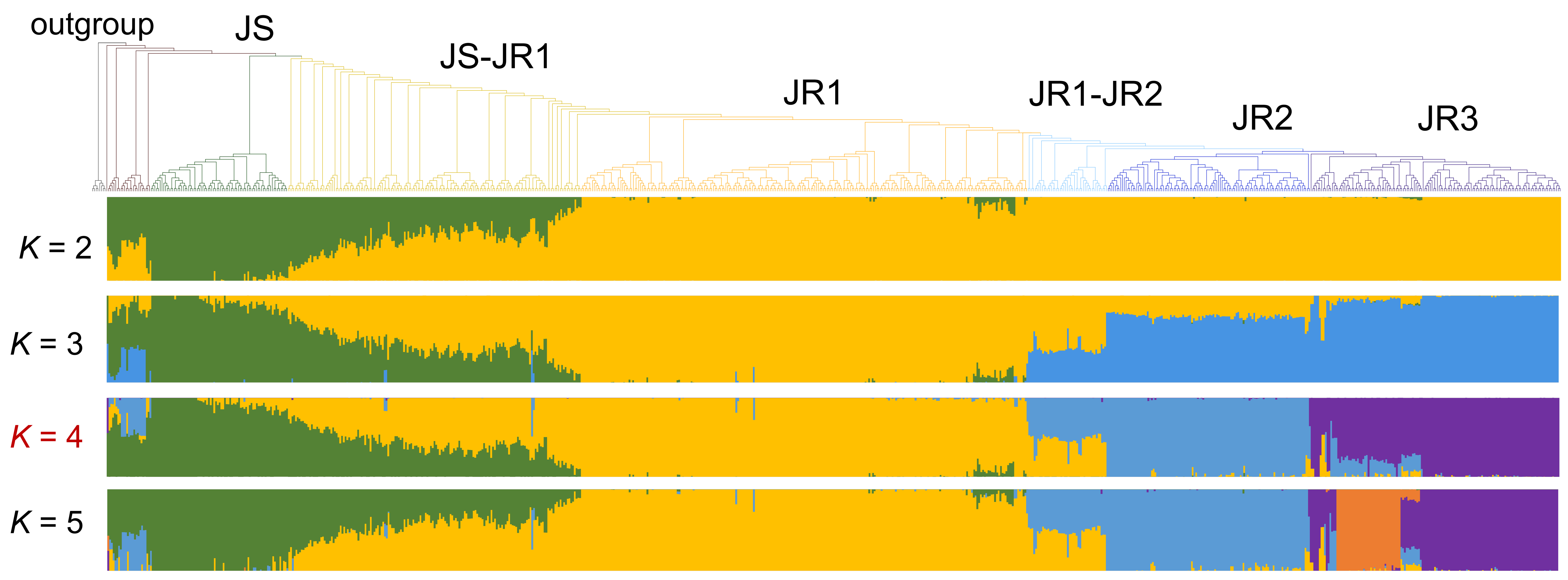

Fig. S2 | Population structure of *J. sigillata* and *J. regia* accessions. The clustering was performed by FastStrucuture with *K* values from 2 to 10. Due to high similarity between individuals, some subgroups could not be clearly determined after *K* = 5. The maximized marginal likelihood value estimated by fastSTRUCTURE was *K* = 4. The order of accessions in the population structure plot is according to the phylogenetic relationship in the tree.

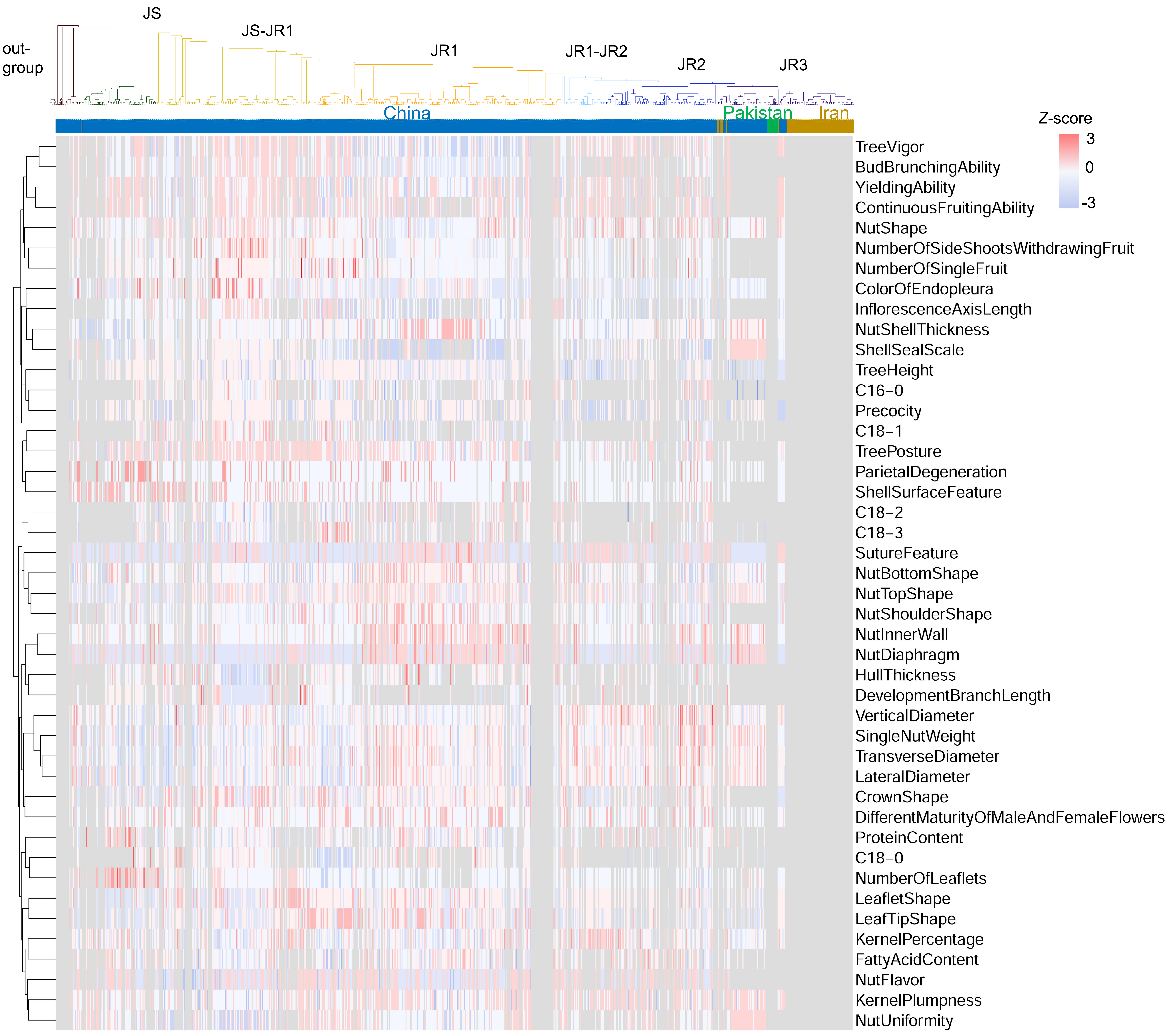

Fig. S3 | Clustering of 44 traits for walnut samples investigated in this study. The normalized phenotypic values of each individual are ordered according to the phylogenetic tree. Since only samples grown in China were phenotyped in this study, the accessions from Iran and Pakistan in JR3 are colored gray.

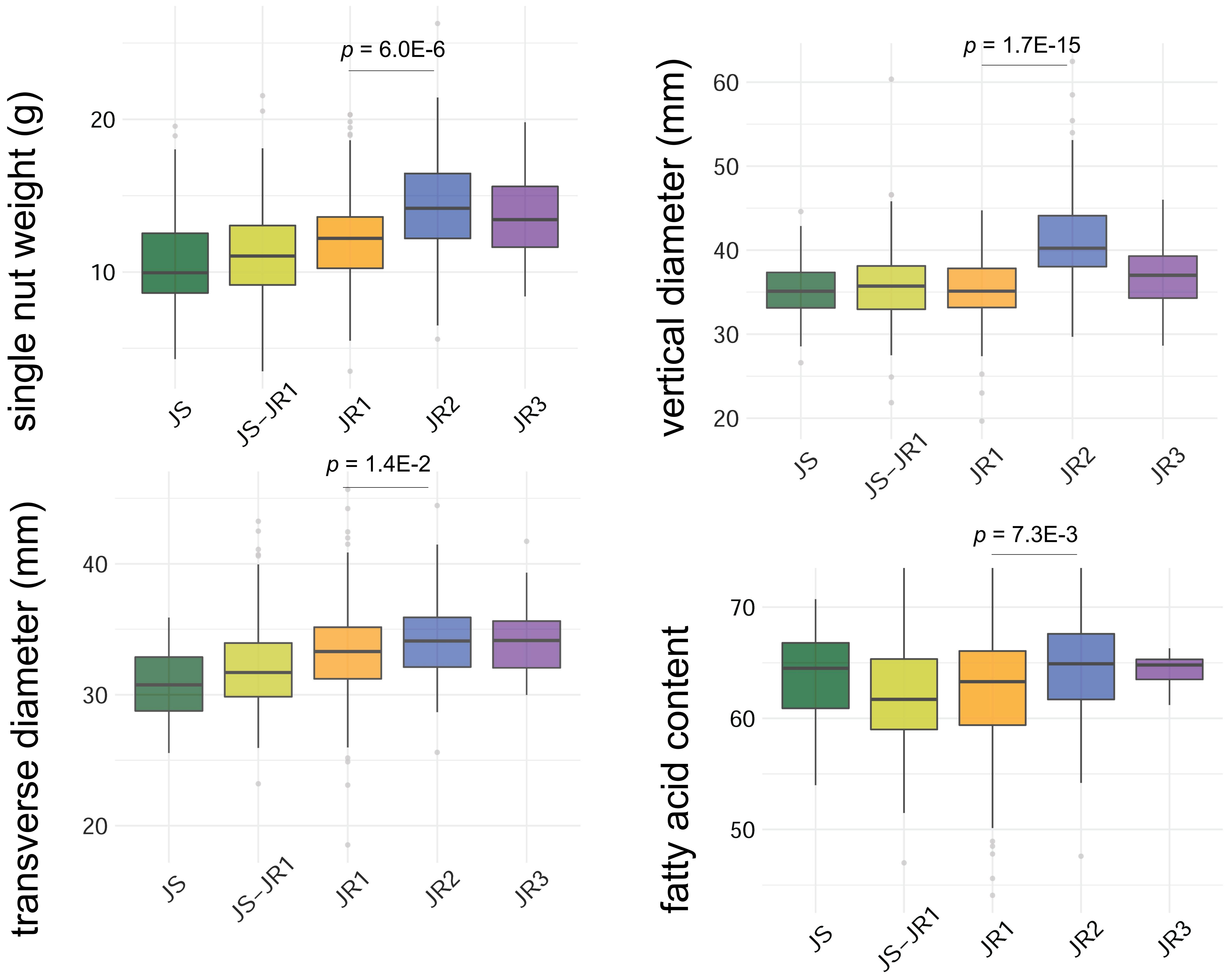

Fig. S4 | Comparison of traits related to walnut fruit weight, size and fatty acid content for different populations. *P* values for the phenotypic differences between naturally evolved population (JR1) and improved population (JR2) are listed (two tailed *t*-test).

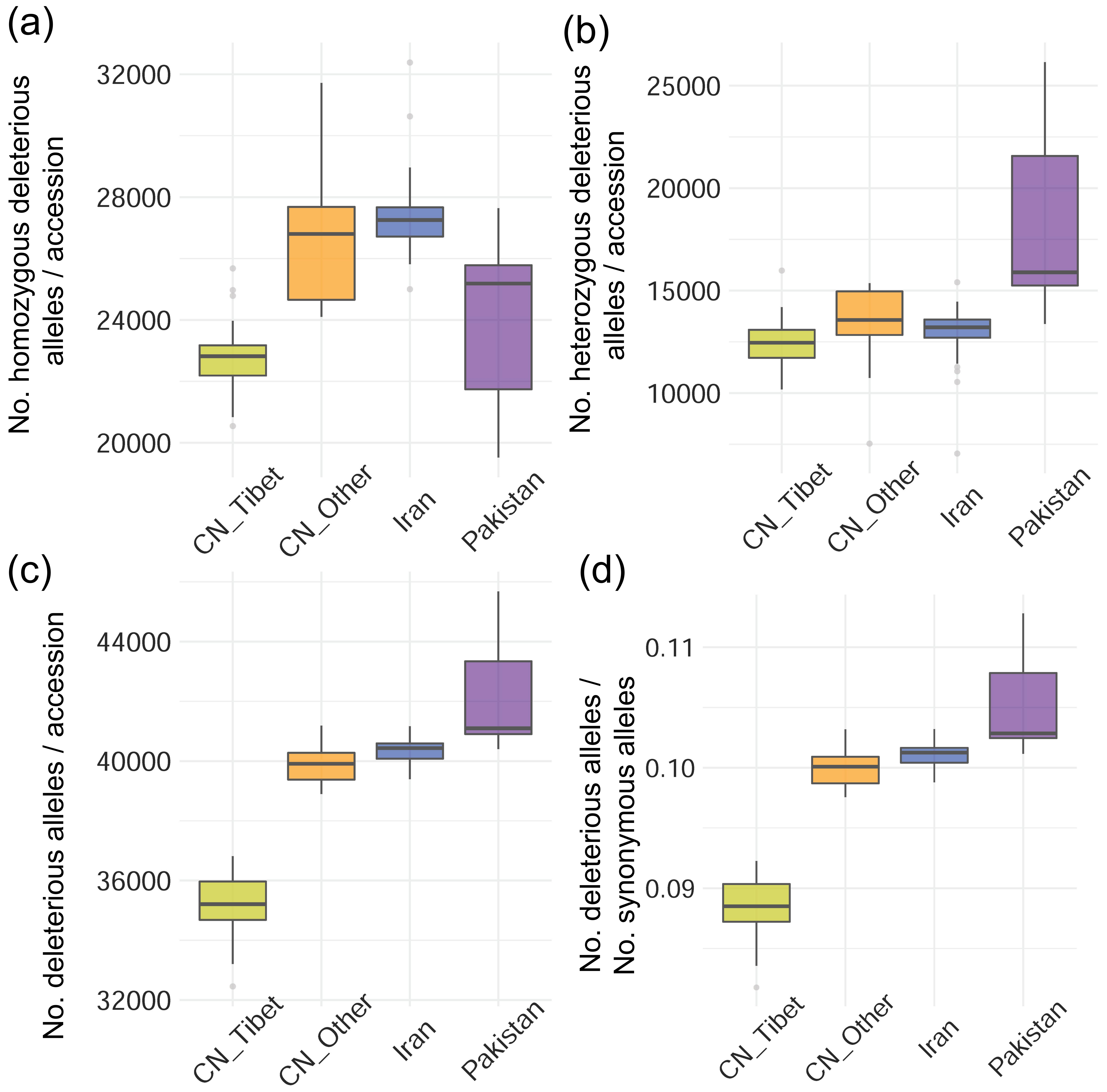

Fig. S5 | Mutation burden for different walnut subpopulations in the JR3 group. The number of homozygous, heterozygous, and total number of deleterious alleles are shown in (a), (b), (c), respectively. The normalized (dSNP/sSNP) mutation burden is shown in (d).

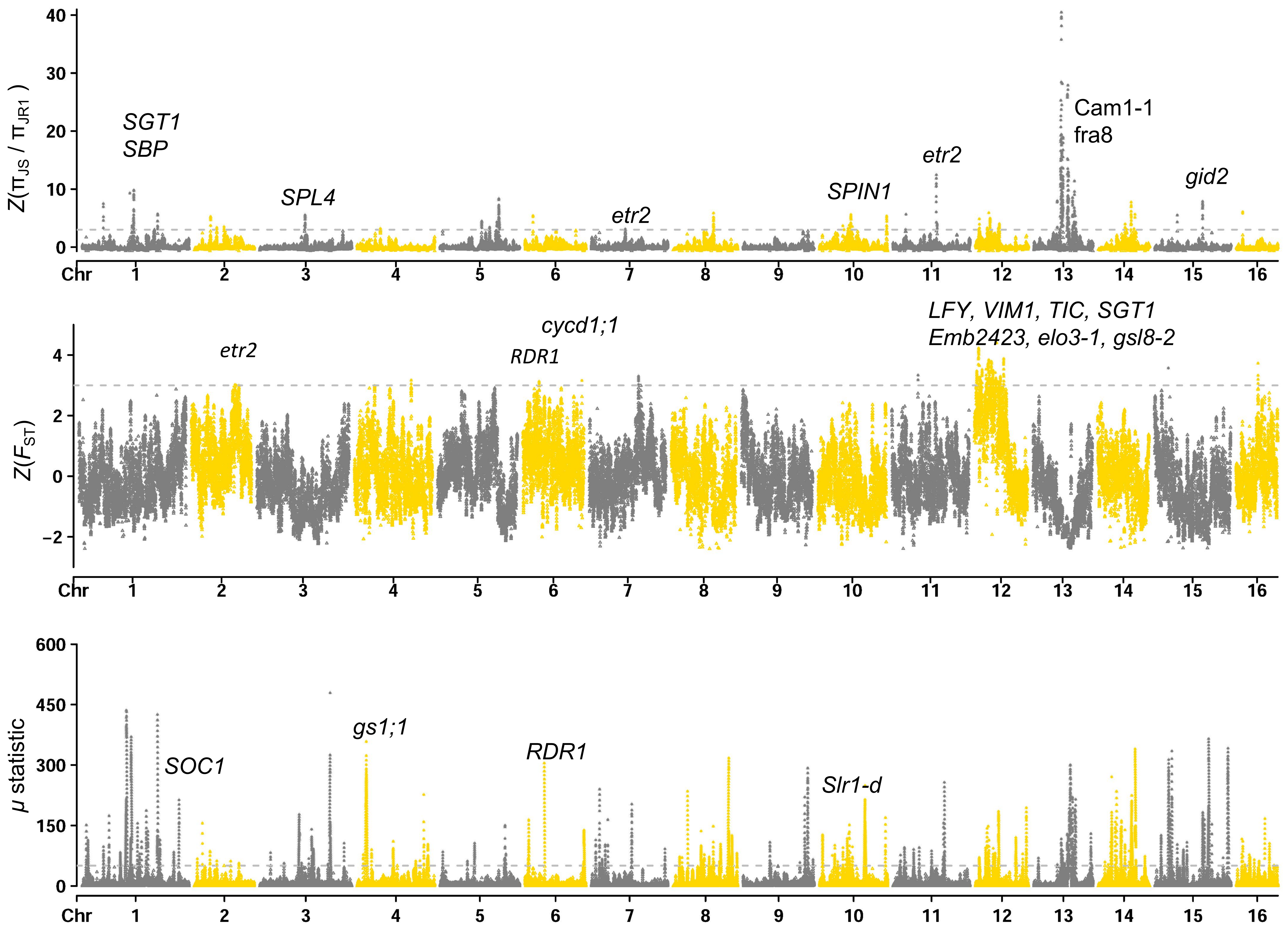

Fig. S6 | Genomic signatures for selection of JR1 walnut population. Selection signatures identified by three approaches are illustrated in the three subfigures, i.e., reduction of genetic diversity (top), genomic differentiation based on $Z(F_{ST})$ (middle), and μ statistics (bottom).

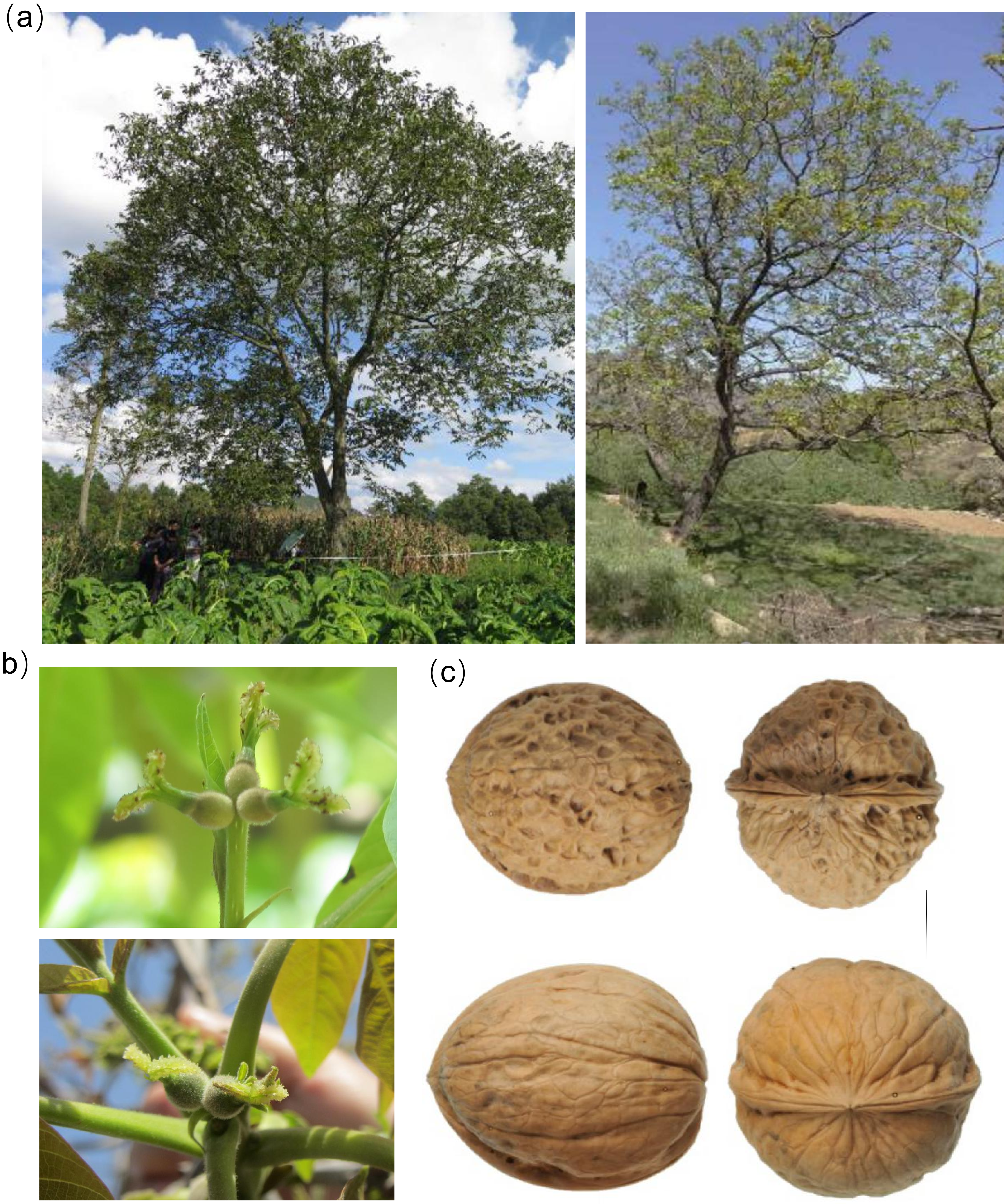

Fig. S7 | Phenotypic differences between *J. sigillata* (JS) and *J. regia* (JR). (a) Tree morphology, left: JS, right: JR; (b) Number of female flowers in each inflorescence; (c) Nut appearance morphology. For b and c subfigures, top: JS, bottom:JR.

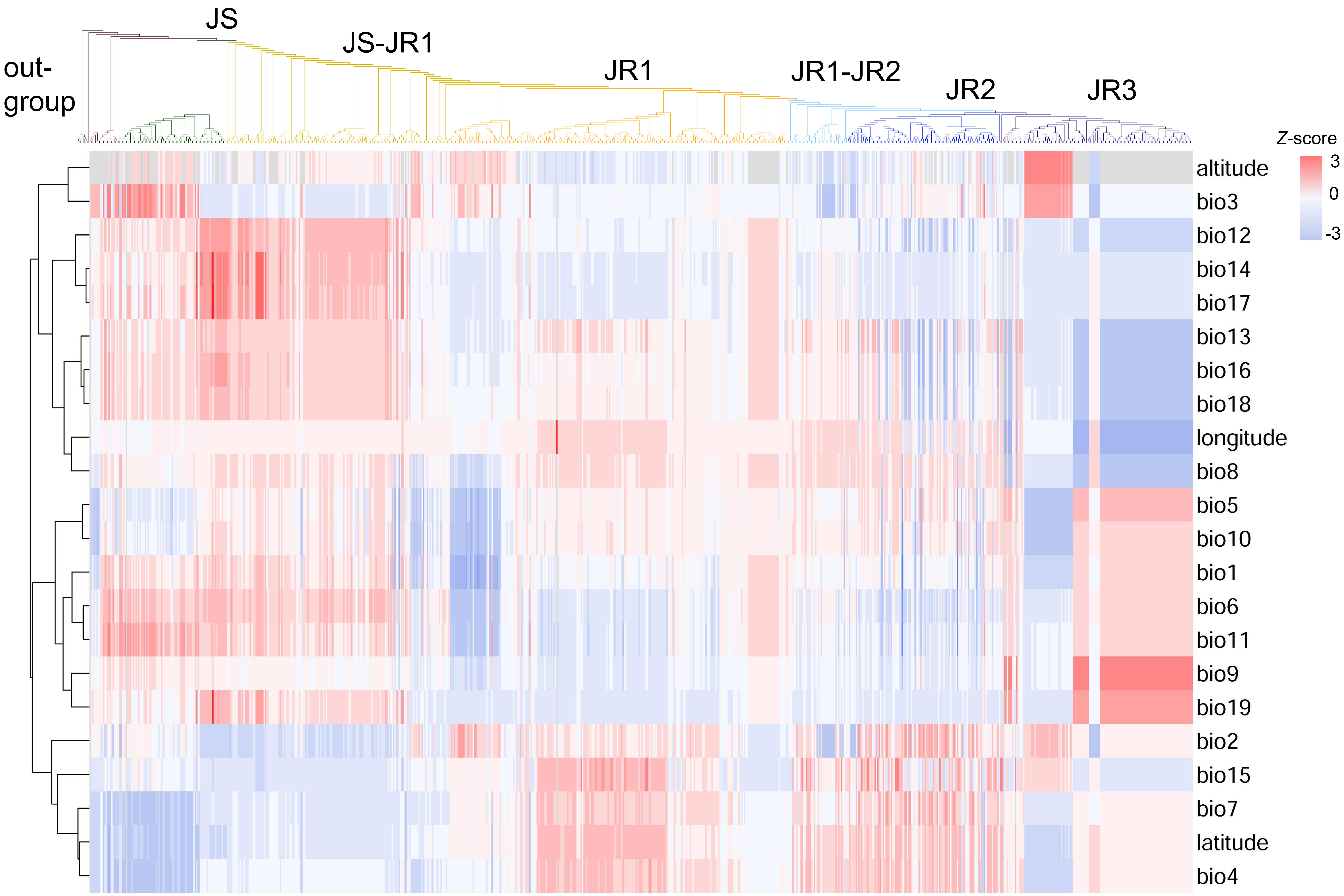

Fig. S8 | Clustering of 20 environmental values for walnut samples investigated in this study. The normalized environmental values of each individual are ordered according to the phylogenetic tree.

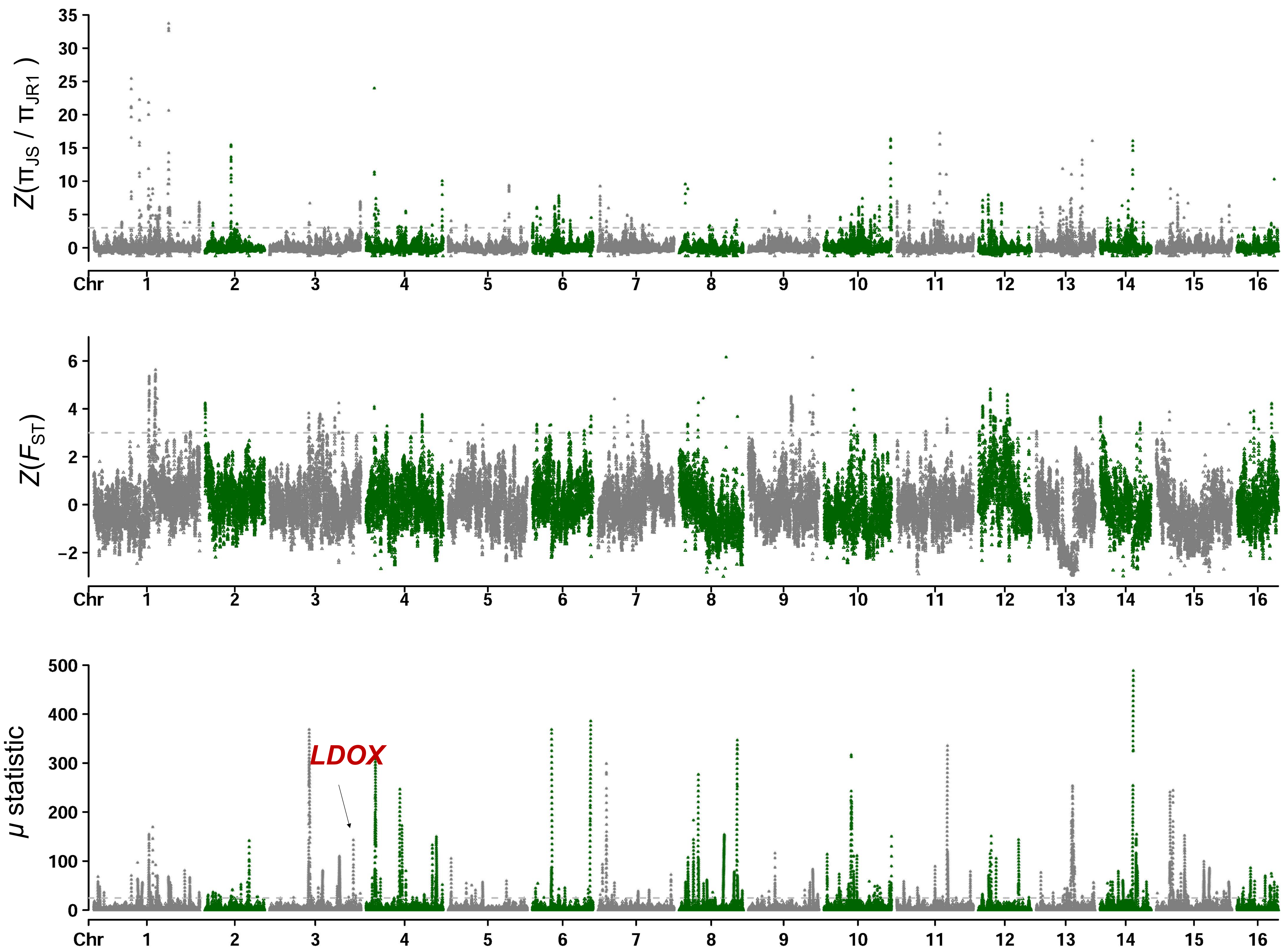

Fig. S9 | Genomic signatures for the Tibetan walnut population. Selection signatures identified by three approaches are illustrated in the two subfigures, i.e., reduction of genetic diversity (up), genomic differentiation based on $Z(F_{ST})$ (middle), and μ statistics (bottom). The *FLS* gene locating in a genomic locus with selection signature is labeled.

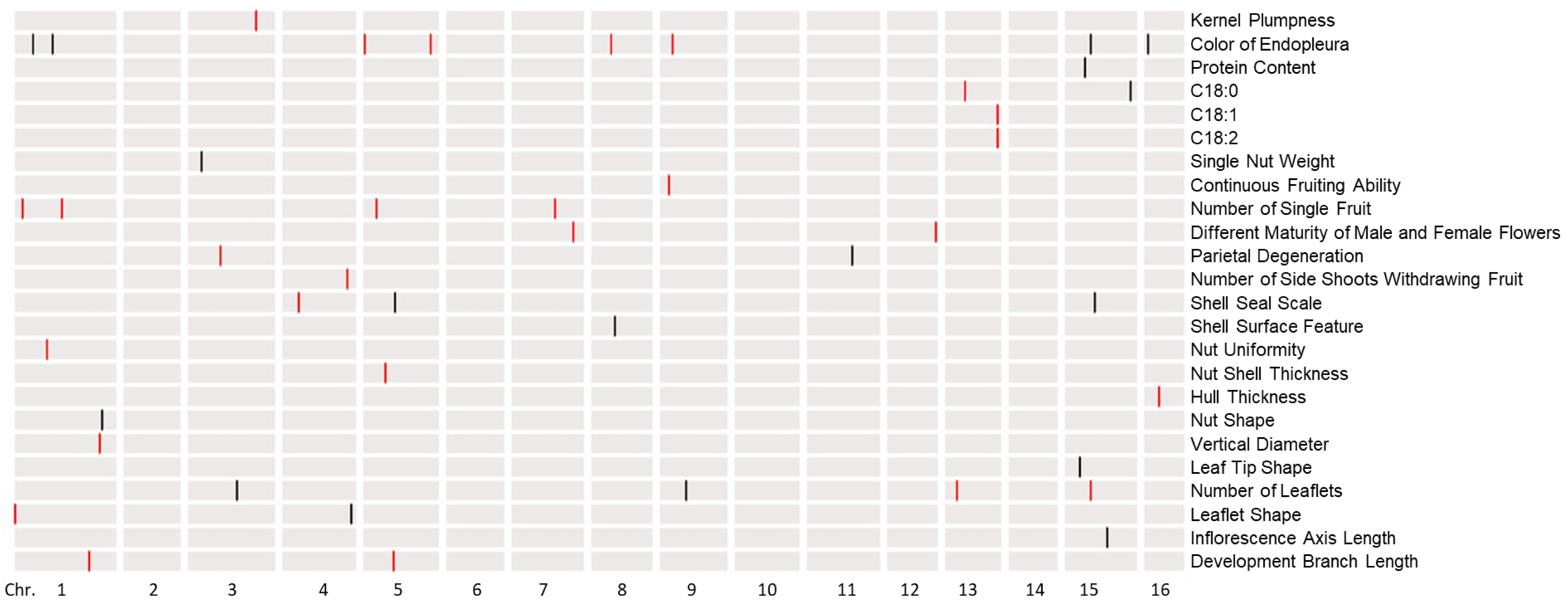

Fig. S10 | Genomic loci associated with agronomic traits of walnut identified by GWAS. All associated loci identified by FastGWA for each trait are indicated in the genome. The red lines indicate that the associated loci by FastGWA are overlapped with those identified by EMMAX software.

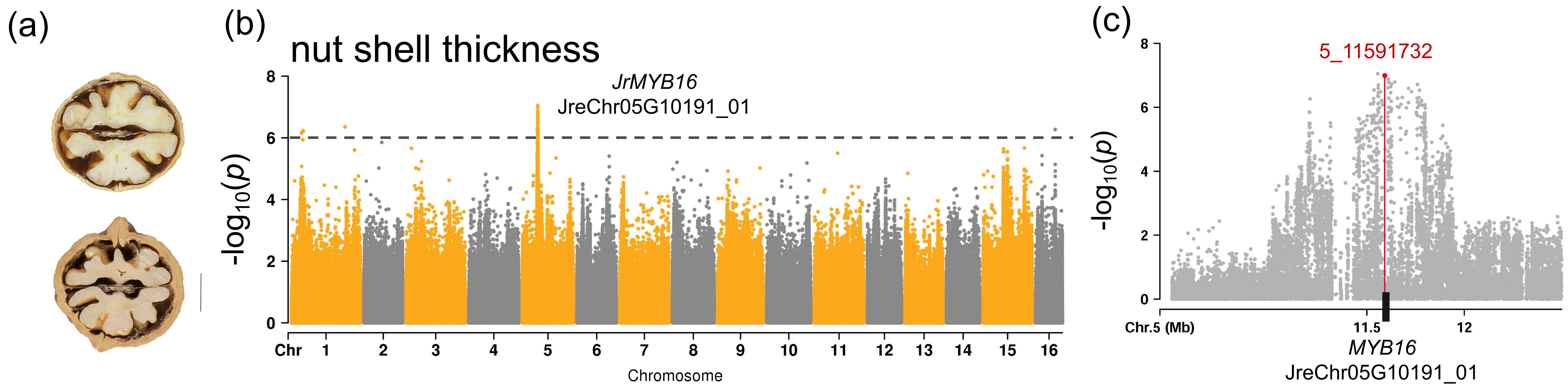

Fig. S11 | Candidate gene associated with nut shell thickness. (a) Phenotypes for thin and thick nut shell are provided. scale bar, 1 cm. (b) Manhattan plot of GWAS for nut shell thickness. The homolog of *MYB16* (JreChr05G10191_01) located in this region is labeled. (c) Zoomed-in plot for the GWAS peak associated with nut shell thickness. One non-synonmynous SNP (5_11591732) in the *MYB16* gene is highlighted.
