# Supplementary material for: A genome variation map provides insights into the genetics of walnut adaptation and agronomic traits
Source: Genome Biol. 2021 Oct 27;22:300. doi: 10.1186/s13059-021-02517-6 (PMC8554829; doi:10.1186/s13059-021-02517-6)
Supplement: Supplementary file 17 — Additional file 17: Supplementary Note. Detailed descriptions for the phenotyped walnut traits. [file 13059_2021_2517_MOESM17_ESM.docx]

**Supplementary Note | Detailed descriptions for the phenotyped walnut traits**


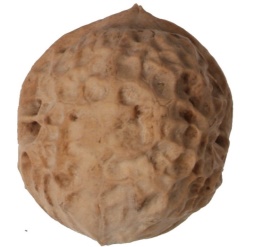

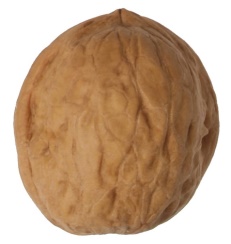


**a**

**b**

**Shell surface feature**: Observe the flatness of the shell surface (the part indicated by the arrow). **a.** ‘1’, Flat; **b.** ‘2’, Concave.


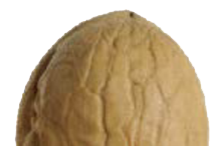

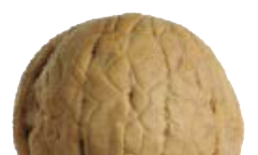

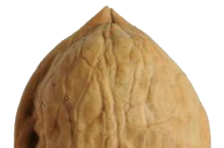


**c**

**b**

**a**

**Nut top shape**: Observe the shape of nut top by visual inspection. The arrow points to the position of the nut top (the location indicated by the arrow). **a.** ‘1’, Sharp; **b.** ‘2’, Slightly sharp; **c.** ‘3’, Flat.


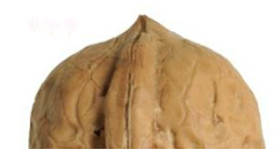

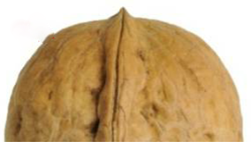

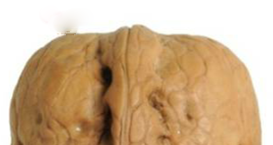

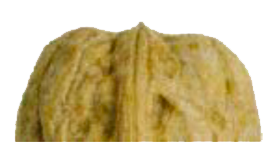


**a**

**b**

**c**

**d**

**Nut shoulder shape**：Observe the shape of the nut shoulder from the side of the suture. The arrow points to the position of the nut shoulder (the location indicated by the arrow).

**a.** ‘1’, Smooth; **b.** ‘2’, Round; **c.** ‘3’, Flat; **d.** ‘4’, Protruding.


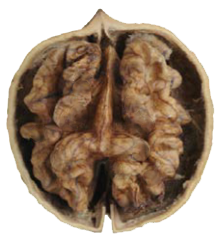

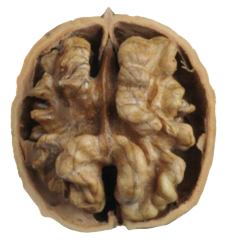

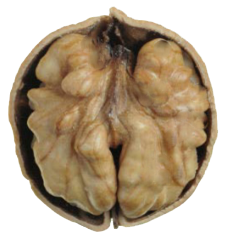


**a**

**b**

**c**

**Kernel plumpness**：Randomly take at least three nuts, peel off the shell, observe the fullness of the kernel by visual inspection.

1. ‘1’, Not full; **b.** ‘2’, Slightly full; **c.** ‘3’, Full.


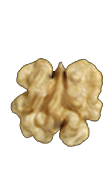

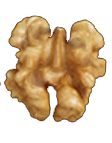

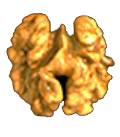

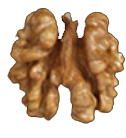

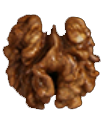

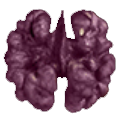

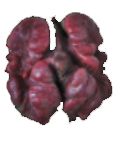

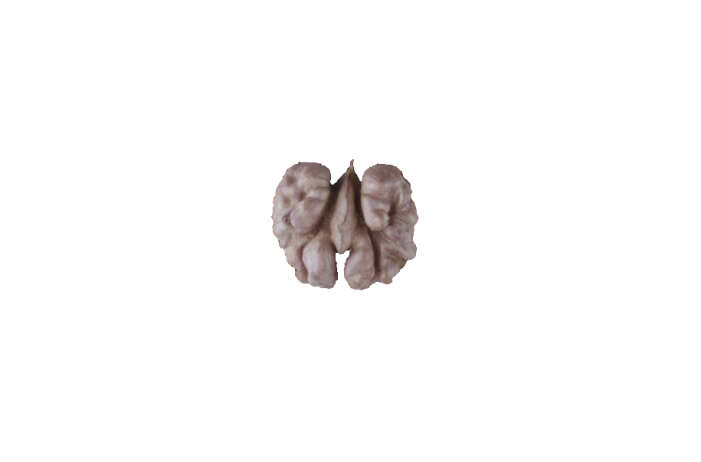


**b**

**a**

**c**

**d**

**e**

**f**

**g**

**h**

**Color of endopleura**: Walnut endopleura colors were categorized into different levels from light to dark. The following phenotypes were determined by visual observation.

**a.** ‘1’, Yellow-white; **b.** ‘2’, Light yellow; **c.** ‘3’, Yellow; **d.** ‘4’, Yellow-brown; **e.** ‘5’, Brown; **f.** ‘6’, Purple; **g.** ‘7’, Purple-red; **h.** ‘8’, Black.


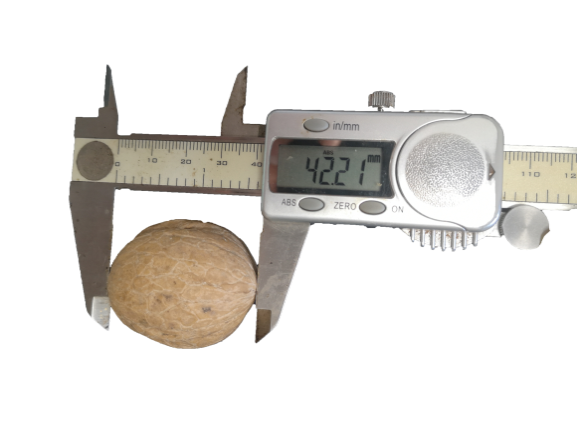


**Vertical diameter (mm)**：The distance between the top and bottom of the walnut nut. Randomly select at least three nuts and measure with vernier calipers.


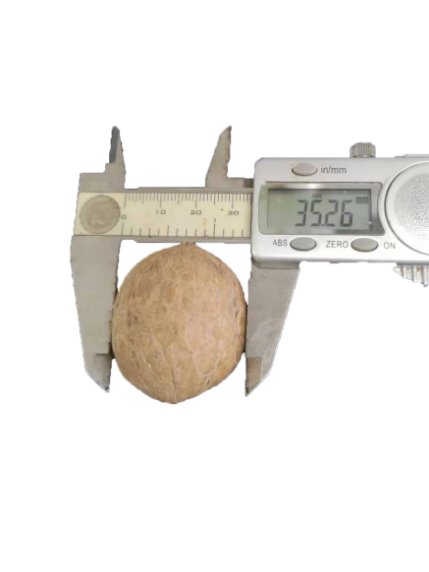


**Transverse diameter (mm)**：The distance between the walnut nut suture lines. Randomly select at least three nuts and measure them with a vernier caliper.


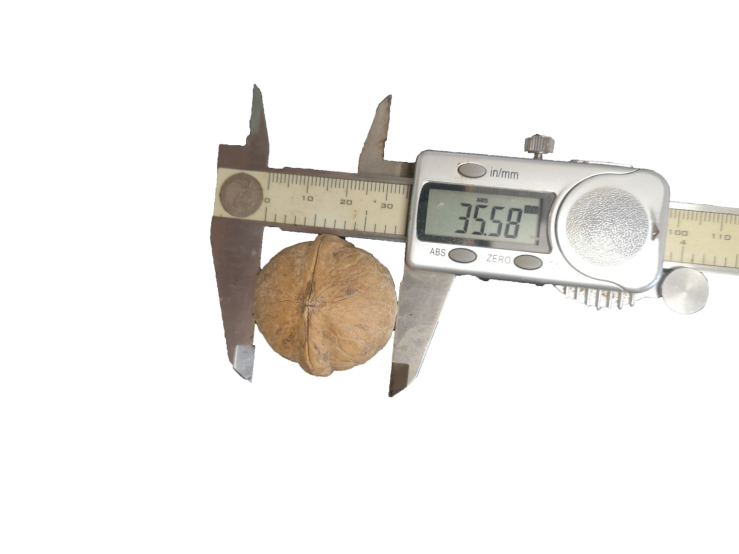


**Lateral diameter (mm)**：The distance between the middle of the walnut nut. Randomly select at least three nuts and measure them with a vernier caliper.


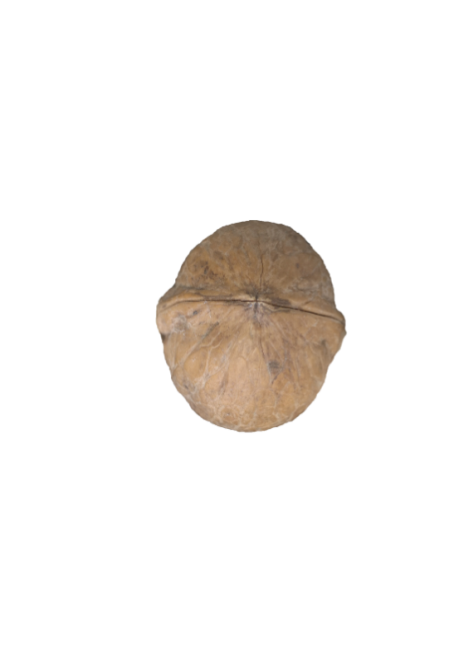

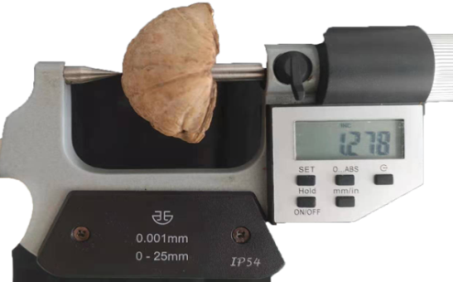


**Nut shell thickness**：Take at least three nuts at random and measure the thickness of nut with a spiral micrometer (the part indicated by the arrow).

‘1’, Incomplete shell (<0.5 mm); ‘2’, Paper shell (0.5~1.0 mm); ‘3’, Thin shell (1.0~1.5 mm); ‘4’, Medium shell (1.6~2.0 mm); ‘5’, Thick shell (2.0~4.0 mm)


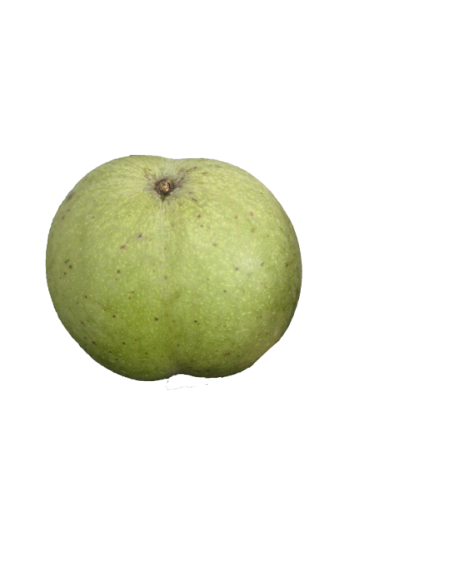

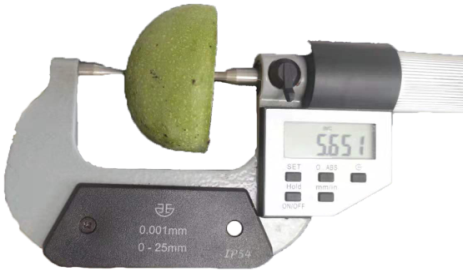


**Hull thickness**：Take at least three walnuts at random and measure the hull thickness with a spiral micrometer.

**Kernel percentage**：Take at least three nuts at random, weigh their total fruit weight and total kernel weight, and calculate the kernel rate according to the following formula：

Kernel Percentage (%) = kernel weight / fruit weight * 100%

**Nut inner wall**：Randomly take at least three nuts, tap the nuts with a small hammer, peel off the nut shell, and observe the characteristics of the nut inner wall between the kernels.

‘1’, Degraded; ‘2’, Leathery; ‘3’, Bony.


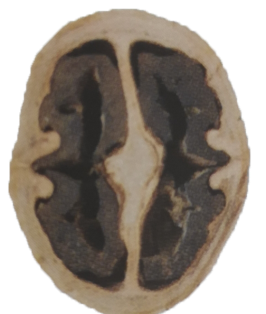

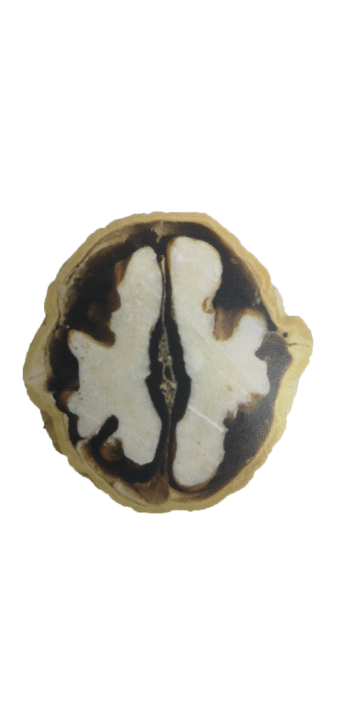

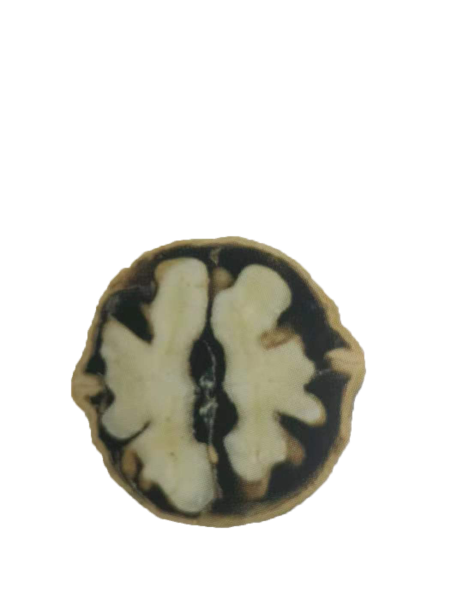


**a**

**b**

**c**

**Nut diaphragm**：Randomly take at least three nuts, tap the nuts with a small hammer, peel off the nut shell, and observe the characteristics of the nut diaphragm between the kernels.

**a.** ‘1’, Membranous; **b.** ‘2’, Leathery; **c.** ‘3’, Bony.

**Nut uniformity**：Take at least three nuts at random, and weigh each nut with a scale.

‘1’, Uneven; ‘2’, Slightly even; ‘3’, Uniform.

**Nut flavor**：Randomly take at least three nuts, take their kernel, and evaluate them in accordance with the relevant part of the General Theory of Sensory Analysis Methodology.

‘1’, Bad; ‘2’, Medium; ‘3’, Good.

**Nut shape**：Take at least three nuts at random and observe the shape of the nuts from the side of the suture.

‘1’, Oblate; ‘2’, Round; ‘3’, Triangle; ‘4’, Broad ovoid; ‘5’, Oval; ‘6’, Wide trapezoid; ‘7’, Trapezoid; ‘8’, Rectangle; ‘9’, Broad oval; ‘10’, Oval; ‘11’, Heart shape; ‘12’, Oblong.


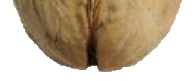

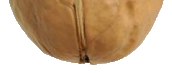

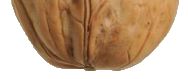

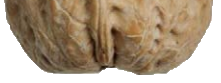


**a**

**b**

**d**

**c**

**Nut bottom shape**：Take at least three nuts at random and observe the shape of the bottom and shoulders of the nuts from the side of the suture.

1. ‘1’, Raised; **b.** ‘2’, Slightly raised; **c.** ‘3’, Flat; **d.** ‘4’, Concave.

**a**

**b**

**c**


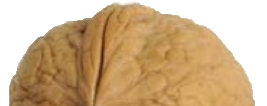


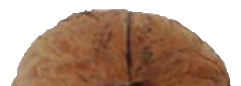

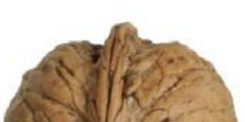


**Suture feature**：Take at least three nuts at random and observe the characteristics of the nut suture by visual inspection.

**a.** ‘1’, Raised; **b.** ‘2’, Flat; **c.** ‘3’, Concave.

**Shell seal scale**：Randomly take at least three nuts, use the walnut suture force test system to determine the tightness of the sutures, the unit is N.

‘1’, Weak (<90 N); ‘2’, Slightly weaker (90~130 N); ‘3’, Medium (130~170 N); ‘4’, Strong (170~300 N); ‘5’, Very strong (>300 N)

**Tree height**：Measure the height of the tree with a pole or height meter.

‘1’, Short (<5.0 m); ‘2’, Medium (5.0~10.0 m); ‘3’, Tall (>10.0 m).

**Tree posture**：Observe the whole morphology of walnut tree plant by visual inspection.

‘1’, Upright; ‘2’, Half open; ‘3’, Open.

**Crown shape**：Observe the crown shape of the walnut tree by visual inspection.

‘1’, Spherosome; ‘2’, Hemispherical; ‘3’, Conical.

**Tree vigor**：Take at least three new treetops outside the canopy to measure their length, and measure the tree vigor with the length of more than 50% of the new treetops.

‘1’, Weak (<20 cm); ‘2’, Medium (20~60 cm); ‘3’, Strong (>60 cm).

**Bud brunching ability**：The ability of the annual shoots to germinate in the spring to extract growing shoots is evaluated as a percentage of the total number of shoots.

‘1’, Weak (<30%); ‘2’, Medium (30%~50%); ‘3’, Strong (>50%).

**Number of side shoots withdrawing fruit**：From the full blooming stage to the fruit maturity stage, randomly select at least three peripheral annual fruiting mother branches as observation objects, and count the number of fruiting branches from each fruiting mother branch's lateral buds.

**Development branch length**：Developmental shoots are more substantial and robust vegetative shoots that have grown from the previous year's leaf buds. During the dormant period, select the annual developmental branches that grow normally outside the crown of the adult tree, and measure the length of the developmental branches with a meter ruler.

**Leaflet shape**：Taking the small leaves in the middle of the mid-pinnate compound leaves of the normally developed branches at the periphery of the crown of the mature fruiting tree as the object, observe the characteristics of the intact small leaves.

‘1’, Oblong; ‘2’, Oval; ‘3’, Broad oval.

**Parietal degeneration**：Take the small leaves at the top of the mid-pinnate compound leaf on the periphery of the crown of the mature fruiting tree as the object, and observe the presence or absence of the top small leaves.

‘1’, Exist; ‘2’, Not exist.

**Number of leaflets**：Taking the mid-pinnate compound leaves of the normally developing branches at the periphery of the canopy as the observation object, select at least three pinnate compound leaves to count the lobules, and calculate the average value, and the unit is slice.

**Leaf tip shape**：Take the middle leaflet on the mid-pinnate compound leaf of the normally developed branch on the periphery of the adult fruiting tree crown as the object to observe the shape of the tip of the intact leaflet.

‘1’, Taper shape; ‘2’, Sharp shape; ‘3’, Blunt shape.

**Different maturity of male and female flowers**：Taking the male and female flowers of the adult fruiting tree as the observation object, observe the opening time of the female and male flowers of the walnut tree by visual observation.

‘1’, Protandrous type（Male flowers open before female flowers）; ‘2’, Male-female type（Male and female flowers bloom almost at the same time）; ‘3’, Protogynous type（Female flowers bloom before male flowers）.

**Precocity**：Visual observation was used to observe the number of years when the seedling walnut trees began to bear fruit.

‘1’, Early fruit（Fruits within 4 years after sowing）; ‘2’, Late fruit（Bear fruit more than 5 years after sowing）.

**Inflorescence axis length**：Measure the length of the inflorescence axis with a vernier caliper, the unit is cm.

**Number of single fruit**：Before the fruit matures, select mature trees and investigate the number of fruit on the outer branches of the canopy.

**Yielding ability**：Calculate the nut yield per square meter of canopy projection area.

‘1’, Low-yield (<0.15 kg); ‘2’, Medium-yield (0.15~0.30 kg); ‘3’, High-yield (>0.30 kg).

**Continuous fruiting ability**: Observe at least three fruiting mother branches, and observe the continuous fruit setting rate according to the distribution of fruit marks on the fruit mother branches.

‘1’, Weak (Can’t bear more than 50% of fruit for two consecutive years); ‘2’, Medium (More than 50% fruit for two consecutive years); ‘3’, Strong (More than 50% of the fruit has been produced for more than three consecutive years).
