# Supplementary material for: A genome variation map provides insights into the genetics of walnut adaptation and agronomic traits
Source: Genome Biol. 2021 Oct 27;22:300. doi: 10.1186/s13059-021-02517-6 (PMC8554829; doi:10.1186/s13059-021-02517-6)
Supplement: Supplementary file 20 — Additional file 20. Review history. [file 13059_2021_2517_MOESM20_ESM.docx]

Review History

**First round of review**

**Reviewer 1**

**Are you able to assess all statistics in the manuscript, including the appropriateness of statistical tests used?**

**Yes, and I have assessed the statistics in my report.**

**Comments to author:**

The manuscript 'Cracking the genetic code for walnut adaptation and agronomic traits: A genome variation map' produced a genomic variation map of 815 walnut accessions. Furthermore, the author uncovered evolutionary history, environmental adaptation and improvement of walnut trees. Candidate genes for economically important traits including linoleic acid content, color of endopleura, and nut shell thickness were further revealed by genome-wide association studies. In general, this research provides a comprehensive genome variation map, and the variations found in walnut will contribute to genetic mechanism study for economically important traits, it deserves publication. However, in the current version of the manuscript, some issues still need to be declared and improved as follows:

1. Line 118 - The accurate rate of SNP and INDEL calling should be validated by experiment, and the loci of false positive should be explained.

2. Line 169 - Do comparison of the genetic diversity characteristics with other perennial tree species, such as tree fruit crops, forest trees.

3. Line 181 - "P-value" should be "P (in italic) value".

4. Line 339 - The correlation and significance of candidate gene expression with phenotypic traits should also be considered and discussed.

5. Line 339 - The authors emphasized the importance of JrFAD2, JrANR and JrMYB16 for controlling linoleic acid content, color of endopleura, and shell thickness of the nut. However, we have not seen the functional verification of any of these genes. In order to provide more evidence to support conclusions, could you provide functional experiments evidence, even in model plants.

6. Line 468 - The authors should give a detailed description of the phenotyping for different traits using Supplementary Note, and the representative phenotype photos need to be attached. In addition, we only found phenotypic data for one year. Usually, two years of phenotypic data are required, especially for the traits you emphasize.

7. Line 552 - The author uses fastGWA for genome-wide association analysis. To improve the accuracy of the results, EMMAX, or TASSEL including appropriate models should also be considered at the same time.

8. Line 554 - "The threshold for significant SNPs is set as 1E-6". Please explain how the significance threshold is obtained.

9. Line 558 - What does bio1-bio19 bioclimatic variables specifically refer to?

10. Line 606 - SNP data sets can provide good resources for the scientific community, and the author need to provide the data in the form of attachments. We have not seen the resequencing data shared by the authors on NCBI, resequencing data with clear ID need to be uploaded.

11. Line 606 - Scripts and pipelines for the study should be provided for other researchers to reproduce the results.

12. Line 616 - There are too many errors in the reference list. Some of them are listed below. Please check carefully by yourself.

1) Ref 3, 6, 31, and so on. The first letter of each word in the title is capitalized, which is inconsistent with other references.

2) Ref 10, 11, and so on. Latin names need to be italicized.

3) Ref 20 "Genome‐ wide selection", Remove extra space.

4) Ref 36, "Bmc"should be "BMC".

**Reviewer 2**

**Are you able to assess all statistics in the manuscript, including the appropriateness of statistical tests used?**

Yes, and I have assessed the statistics in my report.

Comments to author:

This manuscript entitled "Cracking the genetic code for walnut adaptation and agronomic traits: A genome variation map" reported a comprehensive walnut genomic variation map based on the amazing resequencing of 815 representative accessions. By collecting and sequencing representative walnuts, they systematically analyzed the genetic diversity of different walnut populations and explored the characterization of deleterious mutations in walnut genomes. It is interesting that the genetic diversity and heterozygous deleterious mutations of Chinese common walnut trees has continued to increase during the improvement process. Additionally, they measured a total of 46 agronomic traits and identified candidate genes associated with crucial agronomic traits. This is a valuable study and will be of broad interest. The manuscript is globally well written, and I have some concerns with the paper:

1. The short reads mapping ratios of 83, 85, 44, 31, and others are wrong in table S1. They should check these values carefully.

2. Page 5, Line 118; I suggested they provide a figure to show the distributions of 14.78 million SNPs and 3.29 million INDELs on chromosomes.

3. Page 5, Line 119; I suggest they used the number of SNPs and InDels in each Mb to show the densities of SNPs and InDels.

4. It will be better if they can add pictures of representative phenotypes next to the clustered groups in the figure 1a.

5. How many accessions from Iran and Pakistan belong to JR3? I suggest that they highlight accessions from Iran and Pakistan in the phylogenetic tree.

6. Page 6, line 146-147; How to define a variety as an improved variety? This seems strange. Why does the author describe that 39 of the 114 varieties are improved varieties?

7. In Fig. S2, most accessions in JR3 present a uniform gray while analyzing the 46 traits. They should explain why it happens. Also, they used normalized phenotypic values instead of raw values, this should be modified in the description of Fig. S2.

8. They said that JR1 is the naturally evolved population and JR2 is the improved population. I don't know how they distinguish the two populations. They should provide evidence that JR1 is a naturally evolved population and JR2 is an improved population. Why is JR2 not a natural population?

9. In Figure 2a, what are the minimum and maximum values of gene density and repeat density? This information needs to be added.

10. The P-value should be added into Figure 2b.

11. How to define a deleterious allele? And the detailed steps to calculate the homozygous and heterozygous deleterious alleles should be added.

12. From Table S1, I found that most of the accessions come from Iran and Tibet, and it seems that accessions from Tibet are under strong purifying selection. However, in Figure 2b，I found that the difference in the number of mutations is the largest in JR3. I suggest that they further divide JR3 into two subgroups from Tibet and Iran, and calculate the homozygous and heterozygous deleterious alleles.

13. Page 9, line 228; The sentence "Consistent with other plants" is not clear.

14. Page 9, line 228-229; how to define dSNP, tSNP and sSNP? They should explain it.

15. The description of Figure 3 is unclear. For example, what does LoF mean?

16. How to calculate the breathing coefficient, dSNP, tSNP, derived heterozygous SNP? The method part should be improved.

**Authors Response**

**Point-by-point responses to the reviewers’ comments:**

Reviewer #1: The manuscript 'Cracking the genetic code for walnut adaptation and agronomic traits: A genome variation map' produced a genomic variation map of 815 walnut accessions. Furthermore, the author uncovered evolutionary history, environmental adaptation and improvement of walnut trees. Candidate genes for economically important traits including linoleic acid content, color of endopleura, and nut shell thickness were further revealed by genome-wide association studies. In general, this research provides a comprehensive genome variation map, and the variations found in walnut will contribute to genetic mechanism study for economically important traits, it deserves publication. However, in the current version of the manuscript, some issues still need to be declared and improved as follows:

1. Line 118 - The accurate rate of SNP and INDEL calling should be validated by experiment, and the loci of false positive should be explained.

*Reply: We appreciate this suggestion. In the revised version, we performed Sanger sequencing to validate genotypes of 12-19 accessions for 74 SNP sites within 6 genomic fragments (1,232 genotypes in total), and found the overall accuracy of the genotyping rate for the SNPs is 99.0% (added in Additional file 1: Table S2). We noticed that the genotyping accuracy in intergenic regions is lower than that in genic regions. For example, in fragment 4, the genotyping accuracy is 96.7%. In addition, most inconsistently called genotypes between NGS-based bioinformatic approach and Sanger-method are because some homozygous SNPs were mis-called as heterozygous SNPs, which is also noticed in a previous study (Fang et al. 2018). Since all of our subsequent genomic analyses were based on SNPs, only SNP calling results are remained in our revised manuscript.*

*Reference:*

*Fang C, Ma Y, Wu S, Liu Z, Wang Z, Yang R, et al. Genome-wide association studies dissect the genetic networks underlying agronomical traits in soybean. Genome Biol. 2017;18:161.*

2. Line 169 - Do comparison of the genetic diversity characteristics with other perennial tree species, such as tree fruit crops, forest trees.

*Reply: We have taken this helpful suggestion and examined the diversity characteristics in several perennial tree species like peach, pear, tea, jujube based on previous studies (Yu et al., 2018; Li et al., 2019; Wu et al., 2018; Xia et al., 2020; Zhang et al., 2021; Guo et al., 2020) (Table shown below, added in Additional file 1: Table S3).*

*We noticed that unlike common annual crops (e.g. rice, maize, tomato) with consistent reduction of genetic diversity and slower LD decay during domestication and improvement, the genetic diversity of domesticated perennial trees often shows comparable to or even higher than their progenitors. For example, in the tea tree population studies by Xia et al. (2020) and Zhang et al. (2021), the nucleotide diversity (θπ) of cultivated tea plants is higher than their wild ancestors. In addition, cultivated tea trees have a more rapid LD decay than wild populations. For pear trees, as reported by Wu et al. (2018), there are two independent domestication events in Asia and Europe. In European pears, although the θw of wild population (7.38E-3) is much higher than domesticated one (5.21E-3), the θπ value, on the contrary, is slightly higher in the domesticated population. Moreover, Yu et al. (2018) observed a higher genomic heterozygosity rate in improved peach population than its landrace population, which is similar with our walnut findings (i.e., higher genomic heterozygosity in the improved JR2 population). We believe that the higher genetic diversity and genomic heterozygosity in improved walnut population is due to artificial hybridizations during dispersal and immigration during improvement. Also, somatic mutations in the clonally propagated modern walnuts might also lead to elevated population genetic diversity.*

*We have added descriptions related to the genetic diversity comparisons in our revised version of manuscript (Page: 7-8; Lines: 194 - 202).*

*Table | Comparison of population summary statistics in different perennial trees.*

*References:*

*Guo M, Zhang Z, Cheng Y, Li S, Shao P, Yu Q, et al. Comparative population genomics dissects the genetic basis of seven domestication traits in jujube. Hortic Res. 2020;7:89.*

*Li Y, Cao K, Zhu G, Fang W, Chen C, Wang X, et al. Genomic analyses of an extensive collection of wild and cultivated accessions provide new insights into peach breeding history. Genome Biol. 2019;20:36.*

*Wu J, Wang Y, Xu J, Korban SS, Fei Z, Tao S, et al. Diversification and independent domestication of Asian and European pears. Genome Biol. 2018;19:77.*

*Xia E, Tong W, Hou Y, An Y, Chen L, Wu Q, et al. The reference genome of tea plant and resequencing of 81 diverse accessions provide insights into its genome evolution and adaptation. Mol Plant. 2020;13:1013–1026.*

*Yu Y, Fu J, Xu Y, Zhang J, Ren F, Zhao H, et al. Genome re-sequencing reveals the evolutionary history of peach fruit edibility. Nat Commun; 2018;9:5404.*

*Zhang X, Chen S, Shi L, Gong D, Zhang S, Zhao Q, et al. Haplotype-resolved genome assembly provides insights into evolutionary history of the tea plant Camellia sinensis. Nat Genet 2021;53:1250–1259.*

3. Line 181 - "P-value" should be "P (in italic) value".

*Reply: We have modified the format as you suggested throughout the manuscript. Thanks.*

4. Line 339 - The correlation and significance of candidate gene expression with phenotypic traits should also be considered and discussed.

*Reply: We appreciate the reviewer’s insightful suggestion. In the revised version of manuscript, we have investigated the expression profiles of our emphasized genes (JrFAD2 and JrANR) in different tissues and during different developmental stages.*

*In our previous study (Huang et al. 2020), we measured and calculated the accumulation efficiency for linoleic acid (C18:2) and other walnut oil contents during different embryo developmental stages. We found that the biosynthesis efficiency of C18:2 is highest between 91 to 119 days after pollination (Figure JrFAD2 (a)). Based on our early version of manuscript, we found one candidate gene (JreChr15G11197) encoding FAD2 associated with the content of linoleic acid (C18:2) by GWAS analysis. This gene was observed with highest expression value in embryo compared to other eight tissues (Figure JrFAD2 (b)). In addition, in our revised version, through investigating the transcriptomic profiles of this gene at different time points after pollination, we observed the similar pattern between the expression level of the gene and the efficiency of C18:2 biosynthesis (Figure JrFAD2 (c)), which has provided more evidence that the JreChr15G11197 (JrFAD2) as the causal QTG responsible for C18:2 biosynthesis in walnut at transcriptomic level. (Page: 14: Line: 382-389)*

*Figure JrFAD2 | Expression pattern of JreFAD2. (a) Accumulation rate of Linoleic acid (C18:2) and other oil compositions in walnut embryo; (b) Expression level of walnut FAD2 homologs in nine different tissues; (c) Expression levels of JreChr13G11197 (JrFAD2) at different days after pollination.*

*For the candidate gene associated with color of endopleura, based on GWAS, we identified one major locus containing JreChr09G12363 significantly associated with this trait. The JreChr09G12363 is the ortholog of AtANR (BAN) (Figure JrANR (a)), which has been proved to function as a negative regulator of flavonoid biosynthesis and is involved in procyanidin accumulation in the seed (Xie et al. 2003). In our revised version, we examined the transcriptomic profile of this gene in embryo and endopleura, and found a constant and moderate expression level of this gene in embryo during different time points. However, during the development of endopleura, the expression level of JreChr09G12363 (JrANR) had a continuously increasing trend, and FPKM value could reach up to ~800 (Figure JrANR (b)), providing more support for a role of JrANR playing in the endopleura development (Page: 15; Line: 403-408).*

*For the JrMYB16 possibly associated with nut shell thickness, unfortunately, we did not find supportive expression pattern (low expression in our sampled tissues and stages), therefore, we have de-emphasized this gene in our revised manuscript.*

*Figure JrANR | Transcriptomic profiles of ANR homologs in walnut. (a) Identification of ortholog of AtANR in walnut (left). Expression profiles of ANR homologs at different developmental stages of embryo and endopleura in walnut (right). Three replicates for each time point. (b) Expression levels of JreChr09G12363 (JrANR) at different days after pollination.*

*References:*

*Huang R, Zhou Y, Zhang J, Ji F, Jin F, Fan W, et al. Transcriptome Analysis of Walnut (Juglans regia L.) Embryos Reveals Key Developmental Stages and Genes Involved in Lipid Biosynthesis and Polyunsaturated Fatty Acid Metabolism. J Agric Food Chem. 2021;69:377–396.*

*Xie D-Y, Sharma SB, Paiva NL, Ferreira D, Dixon RA. Role of Anthocyanidin Reductase, Encoded by BANYULS in Plant Flavonoid Biosynthesis. Science. 2003;299:396–399.*

5. Line 339 - The authors emphasized the importance of JrFAD2, JrANR and JrMYB16 for controlling linoleic acid content, color of endopleura, and shell thickness of the nut. However, we have not seen the functional verification of any of these genes. In order to provide more evidence to support conclusions, could you provide functional experiments evidence, even in model plants.

*Reply: We highly appreciate your insightful suggestion, and we do agree with you that this work could be greatly improved by adding more functional validations for candidate genes. We have taken your suggestion seriously and strived to verify the biological function of those candidate genes in walnuts or model plants, but unfortunately, up to now we have only got some preliminary results for FAD2. To check the function of FAD2 gene in walnuts, we got JrFAD2 over-expression construct (CaMV35S promoter driven) transformed into somatic embryo of the walnut ‘linzaoxiang’. Firstly, we analyzed the expression of JrFAD2 in 5-week-old somatic embryo from wild-type and two JrFAD2 over-expression line (OE-3 and OE-7). The qRT-PCR results showed that the relative gene expression of JrFAD2 in OE-3 and OE-7 line were 2.1 times and 3.2 times of that in the wild-type, respectively. (Figure 1A). Since FAD2 was reported to catalyze the desaturation of oleic acid to linoleic acid, we analyzed the content of oleic acid and linoleic acid in those somatic embryos. We found that the content of oleic acid (C18:1) and linoleic acid (C18:2) in JrFAD2 over-expression lines significantly decreased and increased compared to wild-type, respectively. (Figure 1B, 1C). These results show that the JrFAD2 probably catalyze the switch of oleic acid to linoleic acid in walnuts. However, we still feel the validation analysis for the JrFAD2 is preliminary (no enough samples for replications) and not very satisfactory, and we decide not include this in our revised manuscript.*

*Functional verification of walnut genes associated with nut traits in vivo is usually tough and too time-consuming due to its long-life circle. In addition, it is also not appropriate to verify some specific nut related traits ectopically in annual model plants. We will be continuing performing comprehensive and solid experimental analysis on the functions of these candidate genes, and hope to report satisfied results in our future studies. For this current work, to our knowledge, it is the first report of the up-to-date most comprehensive walnut genomic resources (e.g., genomic data, variation, association loci for agronomic traits and environmental adaptation), as well as unveiling the population history, and the evolution of walnut deleterious mutations. We do feel this current work could promote walnut improvement and breeding in future, and will also contribute to the nut and perennial fruit tree community.*

*Figure | Over-expression of JrFAD2 Facilitates the Desaturation of Oleic acid to Linoleic Acid. (A) Relative expression levels of JrFAD2 in somatic embryos of wild-type (WT) and JrFAD2 over-expression (OE-3 and OE-7) walnuts. (B), (C) Determination of oleic acid and linoleic acid content in somatic embryos of wild-type (WT) and JrFAD2 over-expression (OE-3 and OE-7) walnuts. **p < 0.01, ***p< 0.001*

6. Line 468 - The authors should give a detailed description of the phenotyping for different traits using Supplementary Note, and the representative phenotype photos need to be attached. In addition, we only found phenotypic data for one year. Usually, two years of phenotypic data are required, especially for the traits you emphasize.

*Reply: Thanks for this helpful suggestion. In our revised manuscript, we have added a supplementary note (Additional file 3: Supplementary Note) describing the traits we phenotyped in detail with related photos attached.*

*In addition, we have also taken your suggestion and added 14 walnut phenotypic data (samples collected and investigated in 2019) into our revised version (Additional file 1: Table S12). We also performed GWAS analyses for these traits by both fastGWA and EMMAX softwares. A total of 21 association signatures for 8 traits could be identified by identified by fastGWA (Additional file 1: Table S15). Among them, 17 associations underlying 7 traits were overlapped with those identified by our first GWAS analyses. For some of interesting walnut traits (e.g., color of endopleura, nut shell thickness) e emphasized in our manuscript, the association signatures showed good consistency.*

*Figure | Comparisons of GWAS results for three traits of two different years.*

7. Line 552 - The author uses fastGWA for genome-wide association analysis. To improve the accuracy of the results, EMMAX, or TASSEL including appropriate models should also be considered at the same time.

*Reply: We have taken your suggestion and also used EMMAX software to perform GWAS for all the 44 traits we phenotyped (we excluded ‘tree age’ and ‘breast diameter’ from the original trait list) in the manuscript. The association signatures identified by fastGWA and EMMAX were compared (Additional file 1: Table S13). The P value threshold was set as 1.16e-6 (details please see our replies for Q#8). Among the 44 associations of 24 traits we identified by fastGWA, 27 associations of 18 traits were overlapped with signatures identified by EMMAX. We do agree with your suggestion that association signatures identified by two methods could improve the accuracy. We have modified the results of this part, and emphasized the overlapped signatures in our revised manuscript. (Page: 13; Lines: 358-366). In addition, the GWAS diagram for the summarized genomic loci associated with walnut traits has been replotted (shown below; Additional file 1: Fig. S10).*

*Figure | Genomic loci associated with agronomic traits of walnut identified by GWAS. All associated loci identified by fastGWA for each trait are indicated in the genome. The red lines indicate that the associated loci identified by fastGWA are overlapped with those identified by EMMAX software.*

8. Line 554 - "The threshold for significant SNPs is set as 1E-6". Please explain how the significance threshold is obtained.

*Reply: In our walnut collection, the total number of individuals with any phenotypic data is 612.*

*A total 6,708,149 SNPs (MAF > 0.05) for 612 accessions with phenotypes were used to perform GWAS analysis. We set the genome-wide significance of all traits with a uniform threshold (p = 1 / the effective number of independent SNPs) (Li et al. 2012). The independent number of SNPs (863,148) was determined by PLINK with the parameters ‘window size = 50, step size = 5, and r2 ≥ 0.5’ (Wang et al. 2016). Therefore, we set the GWAS threshold as 1.16E-6 (1 / 863,148). We have modified the GWAS results based on this threshold.*

*References:*

*1. Wang X, Wang H, Liu S, Ferjani A, Li J, Yan J, et al. Genetic variation in ZmVPP1 contributes to drought tolerance in maize seedlings. Nat Genet. 2016;48:1233–41.*

*2. Li M-X, Yeung JMY, Cherny SS, Sham PC. Evaluating the effective numbers of independent tests and significant p-value thresholds in commercial genotyping arrays and public imputation reference datasets. Hum Genet. 2012;131:747–56.*

9. Line 558 - What does bio1-bio19 bioclimatic variables specifically refer to?

*Reply: We have added the meanings of the bioclimatic variables into Additional file 2: Table S8.*

*The descriptions for the 19 bioclimatic variables are as follows: (from https://www.worldclim.org/data/bioclim.html)*

*bio1 = Annual Mean Temperature*

*bio2 = Mean Diurnal Range (Mean of monthly (max temp - min temp))*

*bio3 = Isothermality (BIO2/BIO7) (×100)*

*bio4 = Temperature Seasonality (standard deviation ×100)*

*bio5 = Max Temperature of Warmest Month*

*bio6 = Min Temperature of Coldest Month*

*bio7 = Temperature Annual Range (BIO5-BIO6)*

*bio8 = Mean Temperature of Wettest Quarter*

*bio9 = Mean Temperature of Driest Quarter*

*bio10 = Mean Temperature of Warmest Quarter*

*bio11 = Mean Temperature of Coldest Quarter*

*bio12 = Annual Precipitation*

*bio13 = Precipitation of Wettest Month*

*bio14 = Precipitation of Driest Month*

*bio15 = Precipitation Seasonality (Coefficient of Variation)*

*bio16 = Precipitation of Wettest Quarter*

*bio17 = Precipitation of Driest Quarter*

*bio18 = Precipitation of Warmest Quarter*

*bio19 = Precipitation of Coldest Quarter*

10. Line 606 - SNP data sets can provide good resources for the scientific community, and the author need to provide the data in the form of attachments. We have not seen the resequencing data shared by the authors on NCBI, resequencing data with clear ID need to be uploaded.

*Reply: Thanks for this suggestion, we have deposited the SNP dataset into the Genome Variation Map (https://ngdc.cncb.ac.cn/gvm/) under the accession number GVM000177.*

*Short-read sequence data of 815 walnut samples generated by this study have been deposited into GenBank under the bioproject accession number PRJNA721107 (SRR14429941 - SRR14430755). The SRR id for each accession has been added into Additional file 1: Table S1.*

11. Line 606 - Scripts and pipelines for the study should be provided for other researchers to reproduce the results.

*Reply: The main scripts for the bioinformatic analyses are also provided in the GitHub repository (https://github.com/lefroyqiu/walnut_pop_genomics_project).*

12. Line 616 - There are too many errors in the reference list. Some of them are listed below. Please check carefully by yourself.

1) Ref 3, 6, 31, and so on. The first letter of each word in the title is capitalized, which is inconsistent with other references.

2) Ref 10, 11, and so on. Latin names need to be italicized.

3) Ref 20 "Genome‐ wide selection", Remove extra space.

4) Ref 36, "Bmc"should be "BMC".

*Reply: We apologize for these errors. We have carefully checked the format of references and fixed them. Thanks!*

Reviewer #2: This manuscript entitled "Cracking the genetic code for walnut adaptation and agronomic traits: A genome variation map" reported a comprehensive walnut genomic variation map based on the amazing resequencing of 815 representative accessions. By collecting and sequencing representative walnuts, they systematically analyzed the genetic diversity of different walnut populations and explored the characterization of deleterious mutations in walnut genomes. It is interesting that the genetic diversity and heterozygous deleterious mutations of Chinese common walnut trees has continued to increase during the improvement process. Additionally, they measured a total of 46 agronomic traits and identified candidate genes associated with crucial agronomic traits. This is a valuable study and will be of broad interest. The manuscript is globally well written, and I have some concerns with the paper:

1. The short reads mapping ratios of 83, 85, 44, 31, and others are wrong in table S1. They should check these values carefully.

*Reply: Thanks for pointing this out. We have modified the reads mapping ratios with the same format (Additional file 1: Table S1).*

2. Page 5, Line 118; I suggested they provide a figure to show the distributions of 14.78 million SNPs and 3.29 million INDELs on chromosomes.

*Reply: We have added one figure showing the distribution of SNPs (Additional file 1: Figure S1). Since all of our subsequent genomic analyses were based on SNPs, only SNP calling results are remained in our revised manuscript.*

3. Page 5, Line 119; I suggest they used the number of SNPs and InDels in each Mb to show the densities of SNPs and InDels.

*Reply: Thanks for this suggestion. We have added this information in the revised manuscript. “For each megabase window across the genome, the average number of SNPs is ~ 30.6k for the 815 walnut individuals collected in this study” (Page: 5; Lines: 121-127). Since all of our subsequent genomic analyses were based on SNPs, only SNP calling results are remained in our revised manuscript.*

4. It will be better if they can add pictures of representative phenotypes next to the clustered groups in the figure 1a.

*Reply: We have added the pictures of nut phenotypes near each group in figure 1a.*

5. How many accessions from Iran and Pakistan belong to JR3? I suggest that they highlight accessions from Iran and Pakistan in the phylogenetic tree.

*Reply: We collected a total of 76 and 11 accessions from Iran and Pakistan, respectively. All of them belongs to JR3. The country origins of the samples have been added in Figure 1a.*

6. Page 6, line 146-147; How to define a variety as an improved variety? This seems strange. Why does the author describe that 39 of the 114 varieties are improved varieties?

*Reply: We apologize for this ambiguity. Among the 728 walnut samples collected in China, 92 accessions were approved as improved varieties by walnut variety approval committees from each province in China. The approved walnut varieties are normally characterized by high yield (e.g., high fruit and nut weight), favorable fruit quality (e.g., light colored kernel, tasty nut), good resistance to pathogens, and are able to adapt well to local agro-ecological conditions. Based on our population structure analysis, there are 114 samples in JR2 group, and 39 of them are improved varieties. We have added more descriptions for the improved varieties in the ‘Plant sampling and DNA sequencing’ of the Methods Section (Page: 18; Lines: 497-502).*

7. In Fig. S2, most accessions in JR3 present a uniform gray while analyzing the 46 traits. They should explain why it happens. Also, they used normalized phenotypic values instead of raw values, this should be modified in the description of Fig. S2.

*Reply: The accessions in JR3 with a uniform gray are from Iran and Pakistan. Only samples grown in China were phenotyped in this study. We excluded ‘tree age’ and ‘breast diameter’ from the original list of 46 traits for GWAS. We have added more clarifications in our revised manuscript (Page: 18; Lines: 509-510).*

*We have modified the description for the supplementary figure: “The normalized phenotypic values of each individual are ordered according to the phylogenetic tree”.*

8. They said that JR1 is the naturally evolved population and JR2 is the improved population. I don't know how they distinguish the two populations. They should provide evidence that JR1 is a naturally evolved population and JR2 is an improved population. Why is JR2 not a natural population?

*Reply: In this study, the phylogenetic and population structure analyses suggested that the JR1 (251 samples) and JR2 (114 samples) could be well distinguished genetically with different ancestries (Fig. 1a). The samples of JR2 showed favorable traits including higher single nut weight, larger nut size, and higher fatty acid content compared to JR1 (Figure shown below; Fig. S4). In addition, as replied in the previous question (#6), we found a much higher proportion of improved walnut varieties in the JR2 (39/114) than in JR1 (14/251). The observation indicated that more artificial selection could have acted on the JR2 population, and JR2 is likely a more derived group compared to JR1. We have removed the term ‘naturally evolved population’ in our revised version.*

*Figure | Comparison of traits related to walnut fruit weight, size and fatty acid content for different populations. P values for the phenotypic differences between JR1 and JR2 are listed (two tailed t-test).*

9. In Figure 2a, what are the minimum and maximum values of gene density and repeat density? This information needs to be added.

*Reply: For estimation of the gene or repeat density of each 1Mb window, we summed up the lengths of genes or repeats within the window, and then divided by the window length. The range for the gene density is 0.016 to 0.568, while repeat density ranges from 0.209 to 0.841. We have added the minimum and maximum values in Figure 2a.*

10. The P-value should be added into Figure 2b.

*Reply: We have added detailed P values into Figure 2b.*

11. How to define a deleterious allele? And the detailed steps to calculate the homozygous and heterozygous deleterious alleles should be added.

*Reply: In this study, we followed previous deleterious variation studies in plants (rice (Liu et al. 2017), grape (Zhou et al. 2017), cassava (Ramu et al. 2017)), and determined the deleteriousness of a SNP mutation site by assessing the degree of sequence conservation (here based on the SIFT software (Vaser et al. 2016)). The detailed steps to identify and calculate the number of homozygous and heterozygous deleterious alleles are as follows:*

*(1) We first generated a combined SNP genotyping matrix including all J. regia and J. sigillata accessions as well as 1 J. nigra (outgroup).*

*(2) The software SnpEff was applied to determine nonsynonymous SNP sites. We further used the SIFT software to calculate the SIFT score, which could estimate the probability of each nonsynonymous SNP to be deleterious (SIFT score < 0.05) or tolerant (SIFT score ≥ 0.05).*

*(3) Since we aimed to calculate the derived deleterious allele for each sample, the genotypes of the J. nigra accession (homozygous state) was used for allele polarization. For a deleterious SNP site, if the SNP genotype of a given sample is homozygous and different from J. nigra, we determined it as homozygous deleterious SNP, then the number of derived deleterious alleles is 2 * No. homozygous deleterious SNPs. If the SNP genotype of a given sample is heterozygous, we determined it as heterozygous deleterious SNP, then the number of derived heterozygous deleterious alleles is 1 * No. heterozygous deleterious SNPs. Finally, the total number of deleterious alleles for each sample is 2 * No. homozygous derived SNPs + 1 * No. heterozygous deleterious SNPs.*

*We have improved the description for these analyses in the Method section (Page: 21-22; Lines: 592-611).*

*References:*

*1. Liu Q, Zhou Y, Morrell PL, Gaut BS. Deleterious Variants in Asian Rice and the Potential Cost of Domestication. Mol Biol Evol. 2017;34:908–924.*

*2. Ramu P, Esuma W, Kawuki R, Rabbi IY, Egesi C, Bredeson JV, et al. Cassava haplotype map highlights fixation of deleterious mutations during clonal propagation. Nat Genet. 2017;49:959–63.*

*3. Zhou Y, Massonnet M, Sanjak JS, Cantu D, Gaut BS. Evolutionary genomics of grape (Vitis vinifera ssp. vinifera) domestication. Proc National Acad Sci. 2017;114:11715–20.*

*4. Vaser R, Adusumalli S, Leng SN, Sikic M, Ng PC. SIFT missense predictions for genomes. Nat Protoc. 2016;11:1–9.*

12. From Table S1, I found that most of the accessions come from Iran and Tibet, and it seems that accessions from Tibet are under strong purifying selection. However, in Figure 2b，I found that the difference in the number of mutations is the largest in JR3. I suggest that they further divide JR3 into two subgroups from Tibet and Iran, and calculate the homozygous and heterozygous deleterious alleles.

*Reply: In this revision, we have taken your helpful suggestion to divide JR3 into different subgroups (samples from Iran, Pakistan, Tibet, and other sample regions in China), and separately calculate different types of genetic burden for them. It is much clearer to see the Tibetan walnuts have the lowest number of deleterious alleles, while higher number of deleterious alleles exists in walnuts from Pakistan. We have added the results in Additional file 2: Fig. S5.*

*Figure | Mutation burden for different walnut subpopulations in the JR3 group. The number of homozygous, heterozygous, and total number of deleterious alleles are shown in (a), (b), (c), respectively. The normalized (dSNP/sSNP) mutation burden is shown in (d).*

13. Page 9, line 228; The sentence "Consistent with other plants" is not clear.

*Reply: We apologize for this ambiguity. The sentence has been rewritten as “We observed that the deleterious SNPs segregated at a lower frequency compared to tolerant and synonymous SNPs in walnut genomes, suggesting a stronger effect of purifying selection acting on the dSNPs than other types of SNPs during walnut evolution” (Page: 9; Lines: 244-246).*

14. Page 9, line 228-229; how to define dSNP, tSNP and sSNP? They should explain it.

*Reply: As replied in question #11, we used SnpEff software to identify nonsynonymous SNPs and synonymous SNPs (sSNP). The SIFT software was further used to classify a nonsynonymous SNP into deleterious SNP (dSNP) or tolerant SNP (tSNP) based on the SIFT score (SIFT < 0.05: dSNP; SIFT ≥ 0.05: tSNP).*

15. The description of Figure 3 is unclear. For example, what does LoF mean?

*Reply: The LoF refers to ‘loss of function’. We assigned the SNPs as ‘LoF’ if the SNPs were annotated as ‘high’ effect (e.g., stop-gains (nonsense), splice site-disrupting SNVs) in the SNPeff software. We added the description in the Figure legend.*

16. How to calculate the breathing coefficient, dSNP, tSNP, derived heterozygous SNP? The method part should be improved.

*Reply: For calculation of dSNP, tSNP, and derived heterozygous SNP, we have replied in question #11.*

*We estimated the inbreeding coefficient for each individual of all walnut samples by VCFtools (Danecek et al. 2011) (https://sourceforge.net/p/vcftools/mailman/message/33590266/). The software estimates the inbreeding coefficient for each individual in the genotyping VCF matrix based on the equation F = (O(hom) - E(hom)) / (N - E(hom)), in which O(hom) is the observed number of homozygous SNPs, E(hom) refers to the expected number of homozygous SNPs, and N is the total number of genotyped loci of the individual. We have added the detailed descriptions and improved our Method section. Thanks for your very helpful suggestions!*

*Reference:*

*Danecek P, Auton A, Abecasis G, Albers CA, Banks E, DePristo MA, et al. The variant call format and VCFtools. Bioinformatics. 2011;27:2156–8.*

**Second round of review**

**Reviewer 1**

The authors addressed all my concerns. I'm satisfied with the revision.

**Reviewer 2**

The revised manuscript has addressed my comments and is much improved. Data analysis became a lot more reproducible as the dataset and pipeline being available and deposited in the GitHub repository. The revised manuscript provides additional transcriptome supports for some of the selected genes which may control important traits for walnut. Here, I still have some comments about this revised manuscript.

1) Confusing interpretation of Demographic history and divergent time estimation. Line 169-173. The authors mainly use SMC++ to estimate the changes of effective population size for JS and JR1 population. However, I found it hard to connect the interpretation in this part with figures. The decreasing timepoint 20-30k generations could not be matched with the figure 1e, as the lines end at around 50k generations. Besides, the estimated divergence time between JS and JR1 seems a little bit weird in the figure and does not have any explanation in the result part.

2) The authors try to use different methods including selective sweeps detection, GWAS, and GEA to detect the potential selected genes for different traits during walnut adaptation. I'm wondering how many genes are correlated given those genes are detected by different selective sweeps methods. Are there many genes that could be co-supported by all of those detection methods, which could turn out to be crucial genes for walnut adaptation and domestication?

3) Figure 6, line 6 under the picture, days after pollination should be abbreviated to DAP not DAF.

4) Page 33, line 923, "cicles" should be corrected into cycles.

5) In Figure S11, there is a spelling mistake in the word synonymous in the last line, please correct it.

**Authors Response**

**Point-by-point responses to the reviewers’ comments:**

Reviewer #2: The revised manuscript has addressed my comments and is much improved. Data analysis became a lot more reproducible as the dataset and pipeline being available and deposited in the GitHub repository. The revised manuscript provides additional transcriptome supports for some of the selected genes which may control important traits for walnut. Here, I still have some comments about this revised manuscript.

1) Confusing interpretation of Demographic history and divergent time estimation. Line 169-173. The authors mainly use SMC++ to estimate the changes of effective population size for JS and JR1 population. However, I found it hard to connect the interpretation in this part with figures. The decreasing timepoint 20-30k generations could not be matched with the figure 1e, as the lines end at around 50k generations. Besides, the estimated divergence time between JS and JR1 seems a little bit weird in the figure and does not have any explanation in the result part.

*Reply: In the Figure for the demographic history (Fig. 1e), the lines for effective population sizes (Nes) end at around 5k generations (not 50k generations). Based on estimations of Nes for JS and JR1 populations, the Ne lines of JR1 reduced sharply relative to those of JS at around 20k to 30k generations ago. Since the split of one population from another is usually accompanied with a sudden reduction in the population size (due to genetic drift), therefore, the divergence time between JR1 and JS is estimated to be around 20k to 30k generations.*

2) The authors try to use different methods including selective sweeps detection, GWAS, and GEA to detect the potential selected genes for different traits during walnut adaptation. I'm wondering how many genes are correlated given those genes are detected by different selective sweeps methods. Are there many genes that could be co-supported by all of those detection methods, which could turn out to be crucial genes for walnut adaptation and domestication?

*Reply: For our detection of selective sweep regions, we applied a combination of three approaches (i.e., Z (πJS / πJR) > 3, Z(FST) > 3, and top 5% for μ statistic) to detect candidate selected genes for JR1 and JR2 both with J. sigillata population as background. The hybrid detection strategy was used in hope to make full use of the advantage of each detection methodology. The IDs of the genes located within any of the identified candidate selective sweep regions were listed in Table S5 and Table S6 with supporting evidences of different approaches.*

*As mentioned in our manuscript, for JR1, we identified 1,961 genes in the selective sweeps by three approaches. Among them, 154 genes could be supported by at least 2 approaches, and only 8 genes could be supported by all 3 approaches (Table S5). For the 8 genes, there are no functional annotations. For the genes with 2 supporting evidences, we could find functional genes involved in important traits like flowering time (TIC, cdc73-2), salinity tolerance (Cam1-1) and dwarfism (gid2) (Table S5).*

*For the JR2 population, a total of 2,002 genes were found in the selective sweep regions identified by any of the three approaches. Among them, 231 and 2 genes could be supported by at least 2 and 3 approaches, respectively. There are 902 genes within the genomic regions specifically selected in the improved walnut JR2 population, among which 96 and 2 genes could be supported by at least 2 and 3 approaches, respectively (Table S6). Based on functional gene annotations, we could find genes homologous to NIT1 (involved in root-growth responses to nitrate and ammonium), DAO (related to seed development), MTP1 (Zn, Cd and Ni translocation), which could be important targets during walnut improvement.*

*In addition, we also tried to examine whether the genes with at least 2 selective sweep evidences could overlap with genes identified by GWAS and GEA. However, unfortunately, we did not find such overlaps. We suppose it could be due to that some adaptive traits (e.g., flowering time, stress tolerance, nutrient use efficiency) were not phenotyped in this study, and these traits are our future focuses. Also, some alleles important to agronomic traits may have not been fully selected for walnut trees at current breeding stage, which calls for great efforts in walnut tree breeding and improvement in future.*

3) Figure 6, line 6 under the picture, days after pollination should be abbreviated to DAP not DAF.

*Reply: The mistake has been modified.*

4) Page 33, line 923, "cicles" should be corrected into cycles.

*Reply: The typo has been corrected. Thanks.*

5) In Figure S11, there is a spelling mistake in the word synonymous in the last line, please correct it.

*Reply: The spelling mistake has been corrected. Thank you.*
